# Intelectin-1 binds and alters the localization of the mucus barrier–modifying bacterium *Akkermansia muciniphila*

**DOI:** 10.1084/jem.20211938

**Published:** 2022-11-22

**Authors:** Juan D. Matute, Jinzhi Duan, Magdalena B. Flak, Paul Griebel, Jose A. Tascon-Arcila, Shauni Doms, Thomas Hanley, Agne Antanaviciute, Jennifer Gundrum, Jessica L. Mark Welch, Brandon Sit, Shabnam Abtahi, Gwenny M. Fuhler, Joep Grootjans, Florian Tran, Stephanie T. Stengel, James R. White, Niklas Krupka, Dirk Haller, Simon Clare, Trevor D. Lawley, Arthur Kaser, Alison Simmons, Jonathan N. Glickman, Lynn Bry, Philip Rosenstiel, Gary Borisy, Matthew K. Waldor, John F. Baines, Jerrold R. Turner, Richard S. Blumberg

**Affiliations:** 1 Division of Gastroenterology, Department of Medicine, Brigham and Women’s Hospital, Harvard Medical School, Boston, MA; 2 Division of Newborn Medicine, Department of Pediatrics, Massachusetts General Hospital, Harvard Medical School, Boston, MA; 3 Institute of Clinical Molecular Biology, Christian-Albrechts-University and University Hospital Schleswig-Holstein, Kiel, Germany; 4 Guest Group Evolutionary Medicine, Max Planck Institute for Evolutionary Biology, Plön, Germany; 5 Institute of Experimental Medicine, Kiel University, Kiel, Germany; 6 Medical Research Council (MRC) Human Immunology Unit, MRC Weatherall Institute of Molecular Medicine, John Radcliffe Hospital, University of Oxford, Oxford, UK; 7 Translational Gastroenterology Unit, John Radcliffe Hospital, University of Oxford, Oxford, UK; 8 The Forsyth Institute, Cambridge, MA; 9 Marine Biological Laboratory, Woods Hole, MA; 10 Division of Infectious Diseases, Brigham and Women’s Hospital, Boston, MA; 11 Department of Microbiology, Harvard Medical School, Boston, MA; 12 Department of Immunology and Infectious Diseases, Harvard T. H. Chan School of Public Health, Boston, MA; 13 Howard Hughes Medical Institute, Boston, MA; 14 Laboratory of Mucosal Barrier Pathobiology, Department of Pathology, Brigham and Women’s Hospital and Harvard Medical School, Boston, MA; 15 Department of Gastroenterology & Hepatology, Erasmus MC, University Medical Center, Rotterdam, Netherlands; 16 Department of Gastroenterology and Hepatology, Amsterdam Gastroenterology Endocrinology and Metabolism & Cancer Center Amsterdam, Amsterdam University Medical Centers, Amsterdam, Netherlands; 17 Resphera Biosciences, Baltimore, MD; 18 Nutrition and Immunology, Technische Universität München, Freising, Germany; 19 Wellcome Trust Sanger Institute, Hinxton, UK; 20 Cambridge Institute of Therapeutic Immunology and Infectious Disease, Jeffrey Cheah Biomedical Centre, and Division of Gastroenterology and Hepatology, Department of Medicine, University of Cambridge, Cambridge, UK; 21 Department of Pathology, Beth Israel Deaconess Medical Center, Harvard Medical School, Boston, MA; 22 Massachusetts Host-Microbiome Center, Department of Pathology, Brigham and Women’s Hospital, Harvard Medical School, Boston, MA

## Abstract

Intelectin-1 (ITLN1) is a lectin secreted by intestinal epithelial cells (IECs) and upregulated in human ulcerative colitis (UC). We investigated how ITLN1 production is regulated in IECs and the biological effects of ITLN1 at the host–microbiota interface using mouse models. Our data show that ITLN1 upregulation in IECs from UC patients is a consequence of activating the unfolded protein response. Analysis of microbes coated by ITLN1 in vivo revealed a restricted subset of microorganisms, including the mucolytic bacterium *Akkermansia muciniphila*. Mice overexpressing intestinal ITLN1 exhibited decreased inner colonic mucus layer thickness and closer apposition of *A. muciniphila* to the epithelial cell surface, similar to alterations reported in UC. The changes in the inner mucus layer were microbiota and *A. muciniphila* dependent and associated with enhanced sensitivity to chemically induced and T cell–mediated colitis. We conclude that by determining the localization of a select group of bacteria to the mucus layer, ITLN1 modifies this critical barrier. Together, these findings may explain the impact of ITLN1 dysregulation on UC pathogenesis.

## Teaser

ITLN1, whose expression is regulated by the unfolded protein response, coats a subset of microorganisms, including *Akkermansia muciniphila*, allows them to thin the inner mucus layer, and increases colitis severity. ITLN1 upregulation in UC may drive similar outcomes.

## Graphical Abstract

**Figure:**
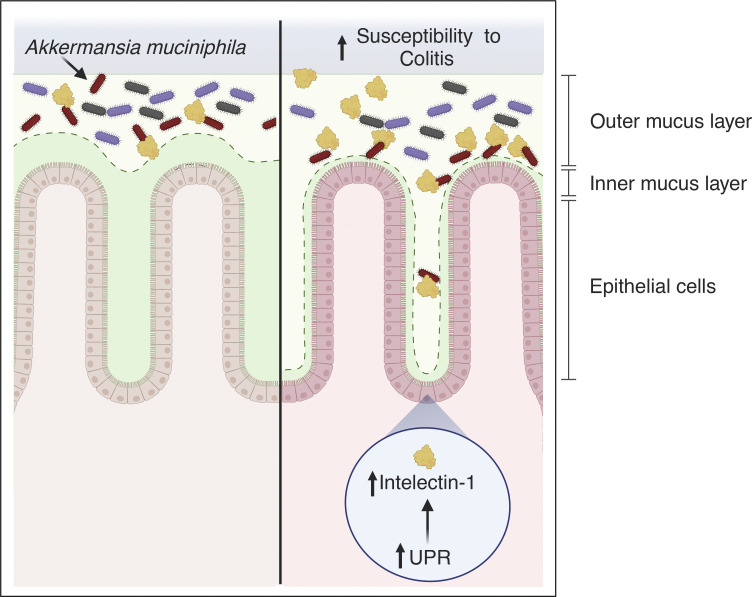

## Introduction

Binding and surveillance of intestinal microbes by secreted host factors are critical for maintaining gut homeostasis. Intelectin-1 (ITLN1) is a lectin produced by intestinal epithelial cells (IECs) and secreted into the intestinal lumen (Parikh et al., 2019; Gremel et al., 2015; Uhlén et al., 2015), where it is part of the core colonic mucus proteome (van der Post et al., 2019). ITLN1 binds to microbial glycans containing terminal exocyclic 1,2-diols and not to mammalian glycans (Wesener et al., 2015; McMahon et al., 2020). *ITLN1* has been identified as a potential genetic risk element for inflammatory bowel disease (IBD; Jostins et al., 2012; Ellinghaus et al., 2016; Huang et al., 2017; Liu et al., 2015); however, the relative contribution of *ITLN1* versus other genes in linkage disequilibrium to this genetic risk locus remains unclear (Nonnecke et al., 2021). Nonetheless, ITLN1 expression is increased in patients with a type of IBD known as ulcerative colitis (UC; Nonnecke et al., 2021), raising the possibility that ITLN1 contributes to UC pathogenesis. Given the microbial binding properties of ITLN1 and the critical role of commensal bacteria and other microorganisms in IBD development (Caruso et al., 2020), a role for ITLN1 in the pathogenesis of IBD is plausible, perhaps by modulating the interaction between intestinal microbes and the mucus. Although the molecular basis of glycan recognition by ITLN1 has been described (McMahon et al., 2020; Wesener et al., 2015), little is known about the spectrum of microbial species recognized by ITLN1 in vivo beyond pathogens in the intestine (Hatzios et al., 2016); furthermore, the impact of these interactions on mucosal homeostasis is unknown.

Transcriptomic and immunohistochemistry surveys have revealed that ITLN1 is expressed in IECs, particularly in goblet cells and Paneth cells in humans and mice, respectively (Haber et al., 2017; Kinchen et al., 2018; Martin et al., 2019; Parikh et al., 2019; Smillie et al., 2019; Wang et al., 2020; Almalki et al., 2021; Nonnecke et al., 2021, 2022). Recent studies suggest that human Paneth cells express mainly ITLN2, unlike mouse Paneth cells that express ITLN1 (Wang et al., 2020; Nonnecke et al., 2021; Nonnecke et al., 2022). Due to their high protein secretory activity, both IEC subtypes are susceptible to ER stress and exhibit elevated unfolded protein response (UPR) activation (Kaser et al., 2008; Deuring et al., 2014). We and others have reported that the UPR is overactivated in IECs in the context of IBD (Kaser et al., 2008; Adolph et al., 2013; Niederreiter et al., 2013; Hosomi et al., 2017; Grootjans et al., 2019; Stengel et al., 2020; You et al., 2021). Here, we report that the UPR is correlated with ITLN1 expression in the intestinal epithelium of patients with IBD and provide evidence that the UPR directly regulates ITLN1 expression at the transcriptional level. Sequencing bacteria bound by ITLN1 in fecal matter revealed that *Akkermansia muciniphila*, a mucin-degrading bacterium (Derrien et al., 2004), was among the subset of bacteria bound by ITLN1 in vivo. This organism has been reported to be protective or deleterious in IBD (Li et al., 2017; Bian et al., 2019; Seregin et al., 2017; Ganesh et al., 2013; Zhang et al., 2021; Kim et al., 2021). To deepen our understanding of the relationship between ITLN1 and IBD, we created a loss of function mouse model (*Itln1*^*−/−*^ mice) and a model with forced expression of ITLN1 under the Villin-1 promoter (*Tg*^*Vil1-Itln1*^ mice), recapitulating the elevated colonic expression of ITLN1 observed in humans with UC.

Overexpression of ITLN1 decreased the thickness of the inner colonic mucus layer and allowed *A. muciniphila* to gain closer access to the epithelial surface of the mucosa under specific pathogen–free conditions (SPF). The phenotype was microbiota dependent as no differences in the inner colonic mucus layer were present among the different genotypes under germ-free conditions (GF). Furthermore, in monocolonization studies with *A. muciniphila*, any expression of intestinal ITLN1 promoted thinning of the inner mucus layer. Overexpression of ITLN1, as occurs in patients with UC, increased the vulnerability of the *Tg*^*Vil1-Itln1*^ mice to chemically induced colitis and T cell–mediated colitis, consistent with the known role of bacterial penetration of the mucus barrier in predisposing to intestinal inflammation (van der Post et al., 2019; Johansson et al., 2008; Johansson et al., 2010; Johansson et al., 2011; Van der Sluis et al., 2006; Vaishnava et al., 2011; Propheter et al., 2017; Brasseit et al., 2016). Lastly, treatment with tetracycline that eradicates *A. muciniphila* (Ansaldo et al., 2019) ameliorated the dextran sodium sulfate (DSS) colitis in *Tg*^*Vil1-Itln1*^ mice. Collectively, these studies show how dysregulation of a specific host protein controlled by the UPR can alter the geographic localization but not the abundance of a specific bacterium. The consequences of aberrant localization of commensal microorganisms in the gut can include diminution of the mucosal barrier and heightened inflammation, contributing to the pathogenesis of IBD.

## Results

### ITLN1 is increased in UC and correlates with the UPR

**Figure 1.**
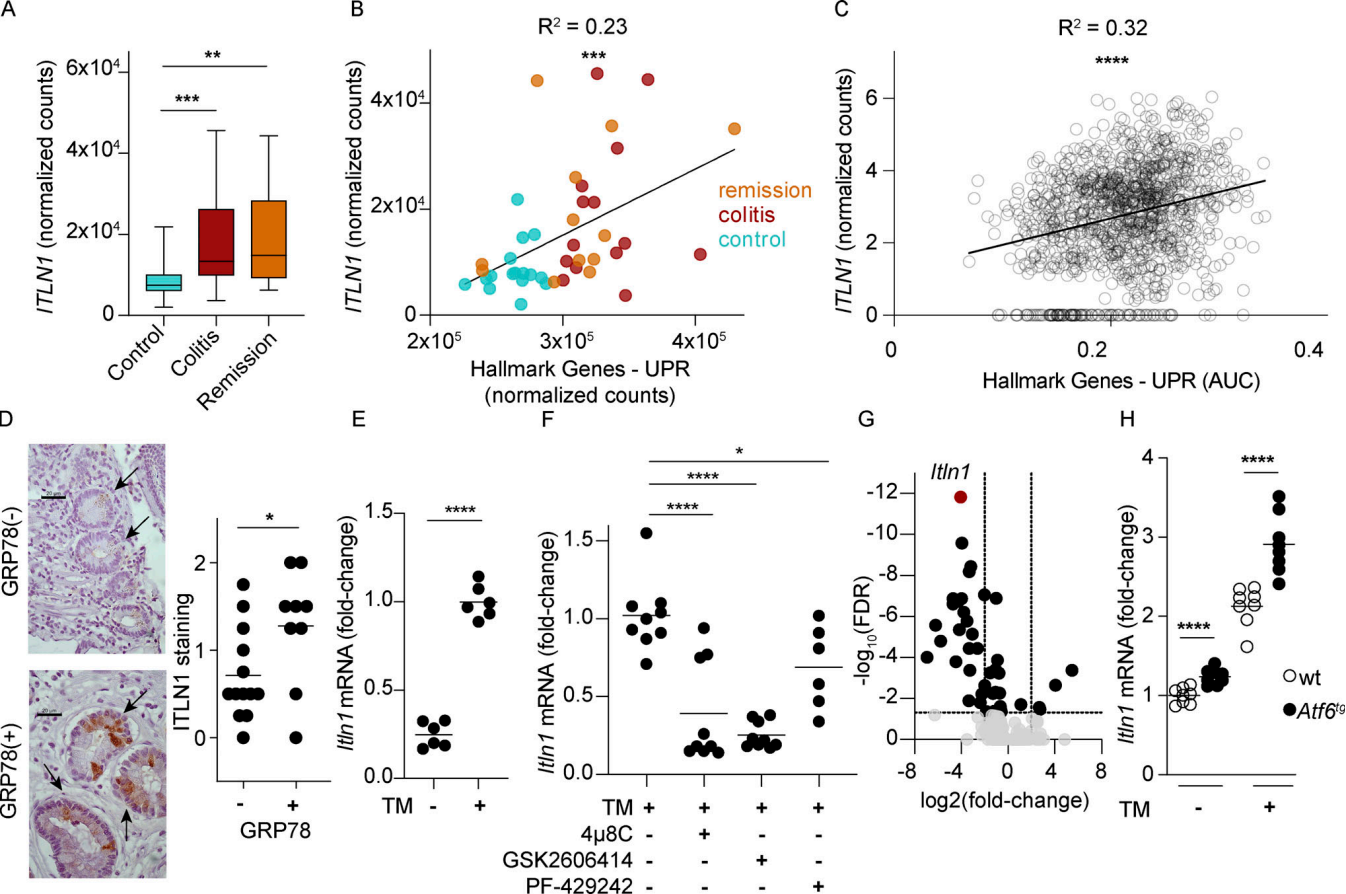
**ITLN1 is increased in response to ER stress in intestinal epithelial cells. (A)** *ITLN1* expression level from bulk RNA-seq performed in colonic biopsies of healthy controls, patients with treatment-naive UC with colitis, or in remission from Taman et al. (2017) (*n* = 14–16). Boxes extend from the 25th to 75th percentile and whiskers from minimum to maximum value, and the line in the middle is the median. **(B)** Correlation between *ITLN1* and UPR hallmark gene expression by bulk RNA-seq in Taman et al. (2017) (*n* = 44). Symbols represent individual human subjects. **(C)** Correlation between UPR hallmark genes and *ITLN1* expression in goblet cells by scRNA-seq from Parikh et al. (2019) (*n* = 1,198 cells). Symbols represent individual epithelial cells. **(D)** ITLN staining in crypts from patients with CD that have GRP78 negative (−) or positive (+) staining of their crypts (*n* = 9–13). Left panel: Representative pictures from small intestine biopsies obtained from GRP78(−) patients and GRP78(+) patients (Deuring et al., 2014). Scale bars indicate 20 µm. Black arrows point to Paneth cells in the crypt. Right panel: Bars represent arithmetic means. Symbols represent individual human subjects. **(E)** Quantification of *Itln1* transcripts by qPCR in mouse small intestinal organoids in the presence or absence of tunicamycin (TM; *n* = 6). Symbols represent individual biological replicates. Bars represent arithmetic means. Data were compiled from two independent experiments. **(F)** Quantification of *Itln1* transcripts by qPCR in mouse small intestinal organoids after TM treatment alone or in the presence of 4μ8c, GSK2606414, or PF-429242 (*n* = 6–9). Symbols represent individual biological replicates. Bars represent arithmetic means. Data were compiled from two to three independent experiments. **(G)** Volcano plot showing log_2_-transformed fold-change of gene expression in crypts obtained by laser capture microscopy from GF *Xbp1*^*ΔIEC*^ mice compared with crypts obtained by laser capture microscopy from GF *Xbp1*^*fl/fl*^ controls (*n* = 3–4). Symbols represent individual genes. **(H)** *Itln1* transcripts by qPCR in intestinal organoids from wild-type and *Atf6*^*tg*^ mice at baseline and after treatment with TM (*n* = 8). Symbols represent individual biological replicates. P values were calculated by Wald-test and corrected for multiple testing by the method of Benjamini and Hochberg (A); generalized linear model (B); generalized negative binomial linear model (C); unpaired *T* test (D, E, and H); one-way ANOVA corrected for multiple comparisons with Dunnet (F); and two-stage step-up method of Benjamini, Krieger, and Yekutieli to control the FDR (G). *P < 0.05; **P < 0.01; ***P < 0.001; ****P < 0.0001.

We first tested if ITLN1 expression was altered in patients with UC using a published bulk RNA-sequencing dataset of mucosal biopsies from treatment-naive patients with UC (Taman et al., 2017). Consistent with a recent report from a different cohort (Nonnecke et al., 2021), *ITLN1* transcripts were increased in biopsies of UC patients regardless of whether the samples were obtained during active disease or remission compared with controls (Fig. 1 A). ER stress and UPR activation are commonly increased in both UC and Crohn’s disease (CD; Kaser et al., 2008; Tréton et al., 2011), prompting us to investigate whether ITLN1 expression correlated with the UPR in humans. *ITLN1* transcript levels were positively associated with UPR hallmark genes (Liberzon et al., 2015) in the human colon (Fig. 1 B). We also found a positive correlation between *ITLN1* transcripts and UPR hallmark genes in goblet cells within our published colonic IEC single-cell dataset (Fig. 1 C; Parikh et al., 2019). Immunohistochemistry of small intestinal crypts from CD patients using a pan-intelectin antibody showed that expression was increased in crypt epithelial cells from patients with positive crypt staining for 78-kD glucose-regulated protein (GRP78^+^) compared with crypt epithelial cells from patients without GRP78 expression in their crypts (GRP78^−^; Fig. 1 D; Deuring et al., 2014). These data suggest that ITLN1 and, perhaps, ITLN2 upregulation are associated with ER stress in intestinal epithelia of IBD patients.

### ITLN1 is regulated by multiple branches of the UPR

**Figure S1.**
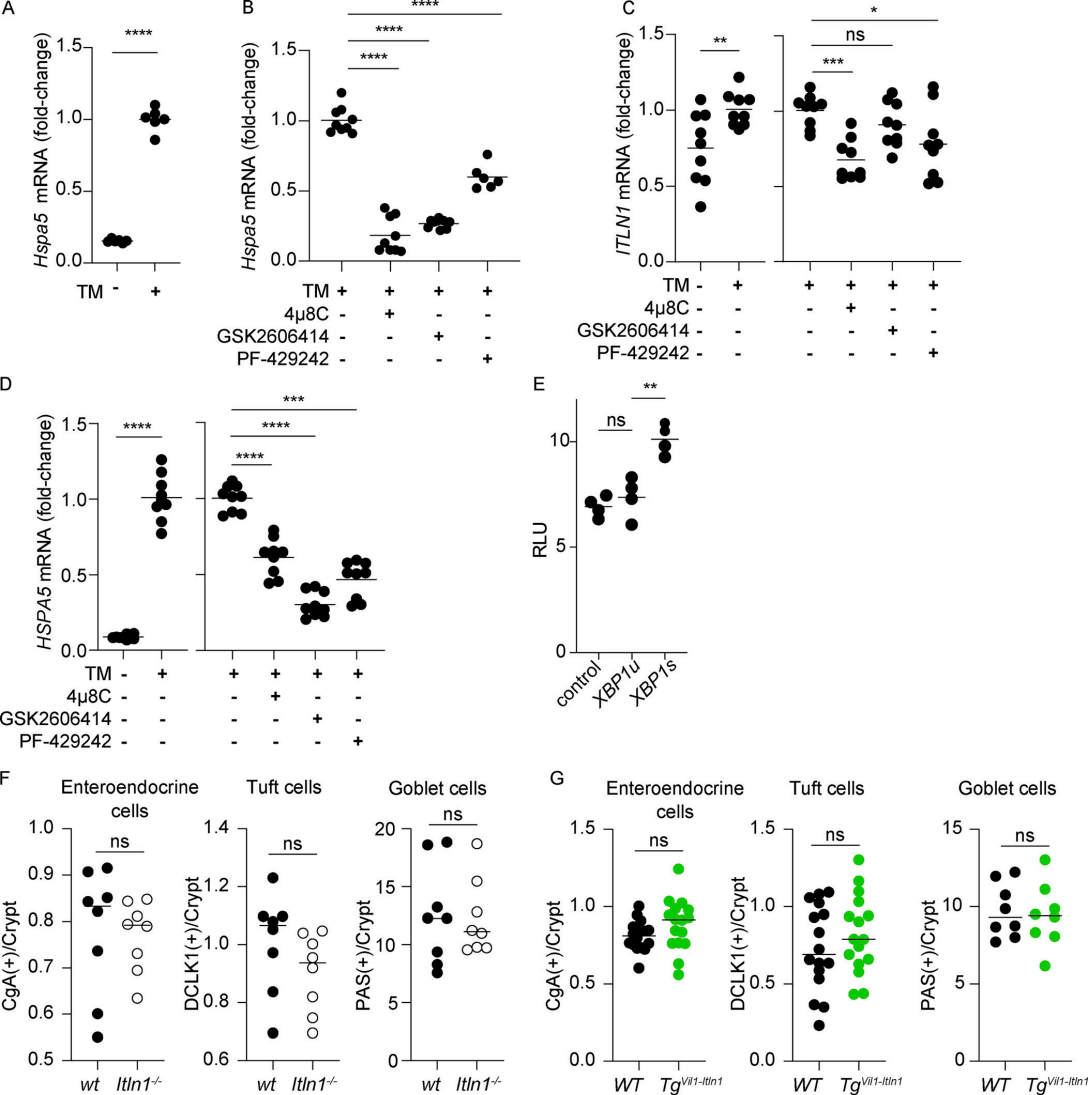
***ITLN1* transcription is induced upon ER stress activation, and its absence or overexpression in intestinal epithelial cells does not affect colonic enteroendocrine cells, Tuft cells, and goblet cells. (A)** Quantification of *Hspa5* transcripts by qPCR in mouse small intestinal organoids in the presence or absence of tunicamycin (TM; *n* = 6). Symbols represent individual biological replicates. Bars represent arithmetic means. Data were compiled from two independent experiments. **(B)** Quantification of *Hspa5* transcripts by qPCR in mouse small intestinal organoids after TM treatment alone or in the presence of 4μ8c, GSK2606414, or PF-429242 (*n* = 6–9). Symbols represent individual biological replicates. Bars represent arithmetic means. Data were compiled from two to three independent experiments. **(C)** *ITLN1* transcripts after tunicamycin treatment alone (TM) or in the presence of 4μ8c, GSK2606414, or PF-429242 in Caco-2 cells (*n* = 9). Symbols represent biological replicate. Bars represent arithmetic means. Data were compiled from three independent experiments. **(D)** *HSPA5* transcripts after tunicamycin treatment alone (TM) or in the presence of 4μ8c, GSK2606414, or PF-429242 in Caco-2 cells (*n* = 9). Symbols represent biological replicate. Bars represent arithmetic means. Data were compiled from three independent experiments. **(E)** Luciferase activity of ITLN1 promoter in HEK293 cells in relative luminescence units (RLU after transfection with *XBP1s*, *XBP1u*, or empty vector [control]; *n* = 4). Symbols represent biological replicate. Bars represent arithmetic means. Data were compiled from two independent experiments. **(F)** Quantification of enteroendocrine cells (Chromogranin A+ cells), Tuft cells (DCLK1+ cells), goblet cells (PAS+ cells) in the colon per crypt of *Itln1*^*−/−*^ mice compared to wild-type littermates (wt; *n* = 8). wt = wild-type littermate from *Itln1*^*−/−*^ colony. Symbols represent individual mice. **(G)** Quantification of enteroendocrine cells (Chromogranin A+ cells), Tuft cells (DCLK1+ cells), goblet cells (PAS+ cells) in the colon per crypt of *Tg*^*Vil-Itln1*^ mice compared to wild-type littermates (WT; *n* = 16). WT = wild-type littermate from *Tg*^*Vil1-Itln1*^ colony. Symbols represent individual mice. P values were calculated by unpaired *T* test (A, C, left panel, D, left panel, F, and G) or one-way ANOVA corrected for multiple comparisons with Dunnet (B, C, right panel, D right panel, and E). *P < 0.05; **P < 0.01; ***P < 0.001; ****P < 0.0001.

We next used in vitro systems to determine whether Intelectin-1 gene transcription is regulated by ER stress in mice and human IECs. *Itln1* transcription was markedly elevated in small intestinal mouse organoids treated with the ER stress–inducing drug tunicamycin (Fig. 1 E), concomitantly with the induction of the UPR marker gene heat shock protein family A member 5 (*Hspa5*), which encodes GRP78 (Fig. S1 A; Stengel et al., 2020). To test which branches of the UPR (Grootjans et al., 2016) mediate increased *Itln1* mRNA expression, we inhibited inositol-requiring enzyme 1α (IRE1α) with 4μ8c (Cross et al., 2012), PKR-like ER kinase (PERK) with GSK2606414 (Guthrie et al., 2016), and activating transcription factor 6α/β (ATF6) by inhibition of site-1-protease with PF-429242 (Lebeau et al., 2018). All branches contributed to the induction of *Itln1* and *Hspa5* as all inhibitors impeded *Itln1* and *Hspa5* transcript upregulation upon tunicamycin treatment (Fig. 1 F and Fig. S1 B). Consistent with these results, *Itln1* transcription was reduced in small intestinal crypt epithelia with conditional knockout of *Xbp1* compared to wild-type littermates (Fig. 1 G). Conversely, *Itln1* transcripts were increased in organoids derived from transgenic mice overexpressing activated ATF6 (*Atf6*^*tg*^; Stengel et al., 2020; Fig. 1 H). We found similar results in the human colonic cell line Caco-2, except for a trend toward inhibition of *ITLN1* transcription upon PERK inhibition that was not statistically significant (Fig. S1, C and D). Lastly, as IRE1α splices *X-Box Binding Protein 1* (*XBP1*) mRNA to become an active transcription factor (*XBP1*s; Grootjans et al., 2016), coexpression of human *ITLN1* promotor (−1.4 KB upstream of the transcription start site) reporter and *XBP1s* in HEK293T cells demonstrated increased transcription relative to cells coexpressing the *ITLN1* reporter and unspliced XBP1 (*XBP1u*; Fig. S1 E). These data suggest that *ITLN1* might be an XBP1 target. These results collectively indicate that *ITLN1* is upregulated in patients with IBD and ER stress and is induced in response to ER stress. These are the first data to link two distinct genetic elements of IBD pathogenesis—ER stress and ITLN1 (Jostins et al., 2012; Ellinghaus et al., 2016; Huang et al., 2017; Liu et al., 2015; Kaser et al., 2008).

### Development of mouse models to study ITLN1 in vivo

**Figure 2.**
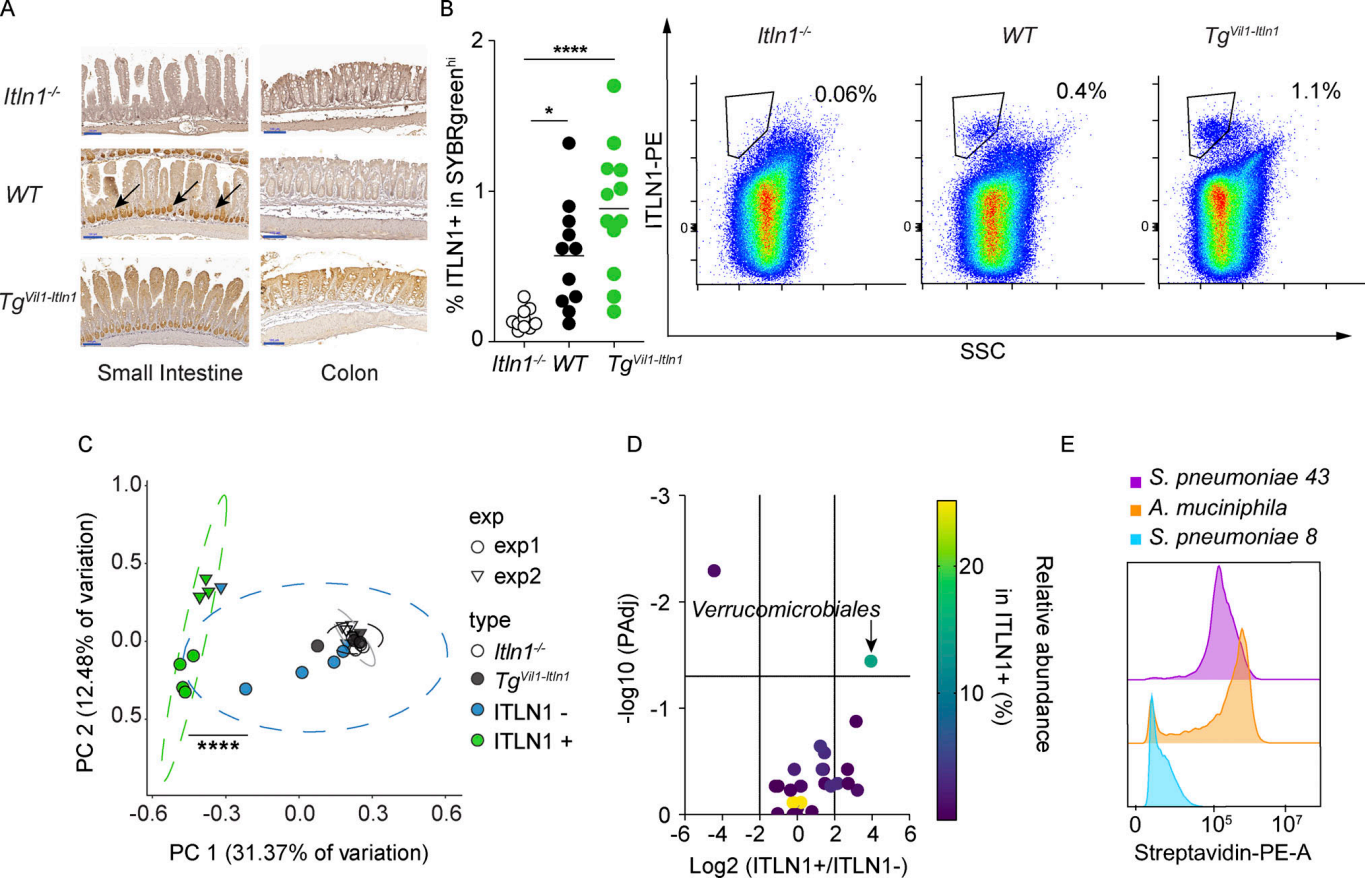
**ITLN1 binds a subset of microbes in the intestinal lumen, particularly *A. muciniphila*. (A)** Representative ITLN1 expression by immunohistochemistry in *Itln1*^*−/−*^, WT, and *Tg*^*Vil1-Itln1*^ mice (*n* = 3). Black arrows point to Paneth cells in the small intestinal crypt of WT mice. Scale bars indicate 100 μm. **(B)** Percentage of bacteria in stools (SYBRgreen^hi^) coated by ITLN1 in *Itln1*^*−/−*^, WT, and *Tg*^*Vil1-Itln1*^ mice with representative density plots gated in SYBRgreen^hi^ (see Fig. S3 Q; *n* = 10–12). Symbols represent individual mice. Bars represent the arithmetic mean. Data were compiled from three independent experiments. **(C)** Principal coordinates analysis (Bray–Curtis dissimilarity) of bacterial communities in *Itln1*^*−/−*^ and *Tg*^*Vil1-Itln1*^ stools before sorting, ITLN1 bound (ITLN1+) and ITLN1 unbound (ITLN1−) fraction post-sorting from *Tg*^*Vil1-Itln1*^ stools. *n* = 7 pooled stools from three mice per genotype. Experiment 1 pooled from same three mice on different days. Experiment 2 pooled stools from different three mice on different days. Symbols represent experiments performed on different days. **(D)** Differential microbial composition of ITLN1+ and ITLN1− fractions. The graph depicts the average log_2_ ratio of relative abundances between ITLN1+ and ITLN1− fractions for each order on the x axis, the corresponding P-adjusted value on the y axis, and the relative abundance in the ITLN1+ fraction depicted by the color bar. *n* = 7 pooled stools from three mice per genotype per replicate. Experiment 1 pooled stools from the same three mice on different days. Experiment 2 pooled stools from three different mice on different days. **(E)** Representative histogram showing binding of human recombinant ITLN1 to *A. muciniphila* isolated from humans in orange, negative control in blue (*Streptococcus pneumoniae* Serotype 8), and positive control in purple (*Streptococcus pneumoniae* Serotype 43; *n* = 3). P values were calculated by one-way ANOVA corrected for multiple comparisons with Dunnet (B); PERMANOVA among ITLN1− and ITLN1+ fraction (C); Welch’s *t* test with log-transformed values in a generalized linear model adjusting for the paired design and two-stage step-up method of Benjamini, Krieger, and Yekutieli to control the FDR (D). *P < 0.05; ****P < 0.0001.

**Figure S2.**
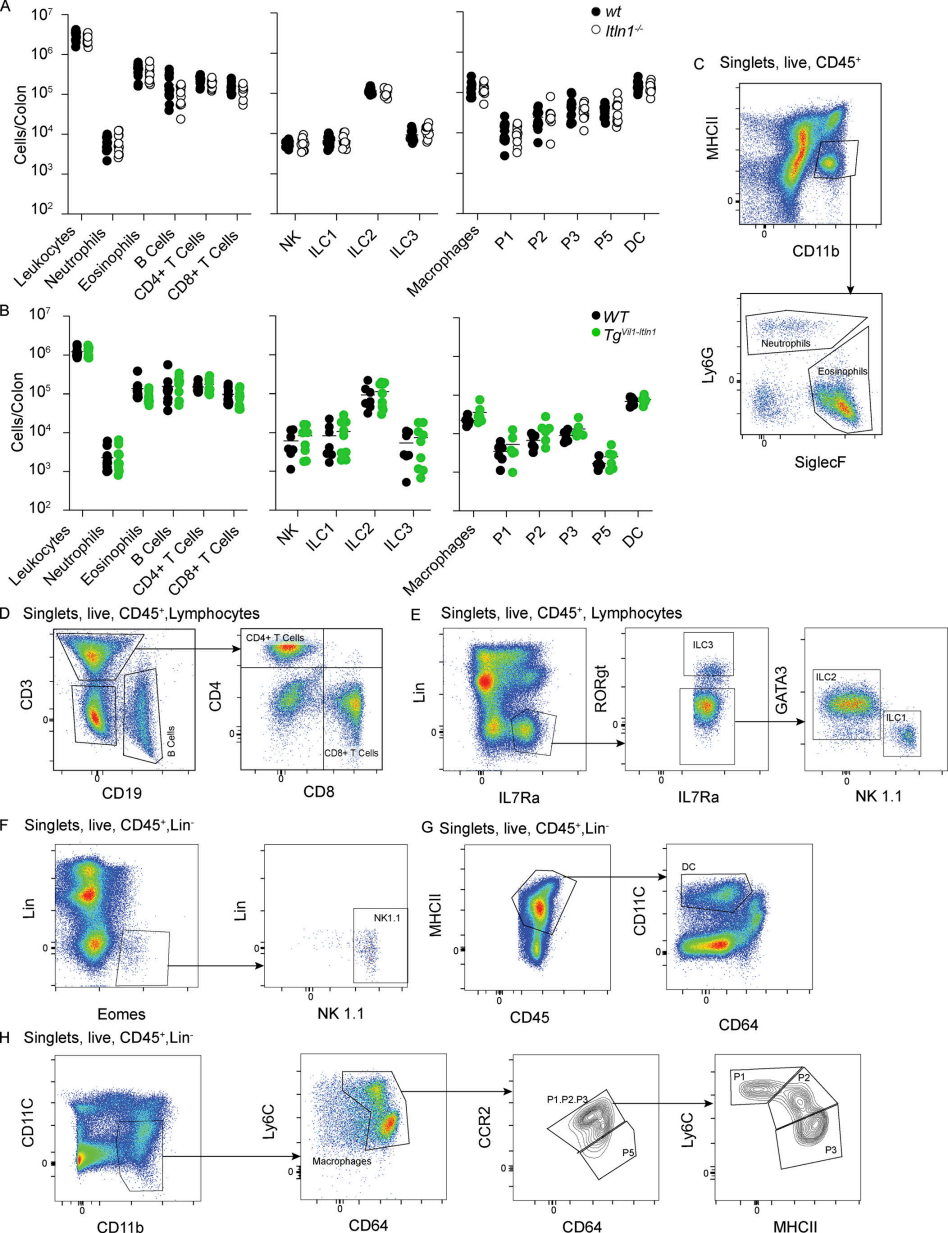
**Baseline characterization of LP leukocytes in the *Itln1***^***−/−***^ **and *Tg***^***Vil-Itln1***^ **mice. (A)** Quantification of leukocytes in colonic LP of *Itln1*^*−/−*^ mice compared to wt littermates (*n* = 11–19). Data were compiled from three independent experiments for the left and right panels and two independent experiments for the middle panel. Symbols represent individual mice. **(B)** Quantification of leukocytes in colonic LP of *Tg*^*Vil1-Itln1*^ mice compared to WT littermates (*n* = 6–11). Data were compiled from two independent experiments for the left and middle panels. Symbols represent individual mice. **(C)** Gating strategy for LP neutrophils and eosinophils in A and B. **(D)** Gating strategy for LP CD4^+^ T cells, CD8^+^ T cells, and B cells in A and B. **(E)** Gating strategy for innate lymphocytes (ILC) 1, 2, and 3 in A and B. Lin = CD3^+^, CD5^+^,CD19^+^, and LY6G^+^. **(F)** Gating strategy for natural killer cells (NK) in A and B. Lin = CD3^+^, CD5^+^, CD19^+^, and LY6G^+^. **(G)** Gating strategies for LP dendritic cells (DC) in A and B. Lin = CD3^+^, NK1.1^+^, CD19^+^, Ly6G^+^, and SiglecF^+^. **(H)** Gating strategies for LP macrophages in A and B. P1, P2, P3, and P5 macrophages correspond to macrophage subpopulations as described in Tamoutounour et al. (2012). Lin = CD3^+^, NK1.1^+^, CD19^+^, Ly6G^+^, and SiglecF^+^.

**Figure S3.**
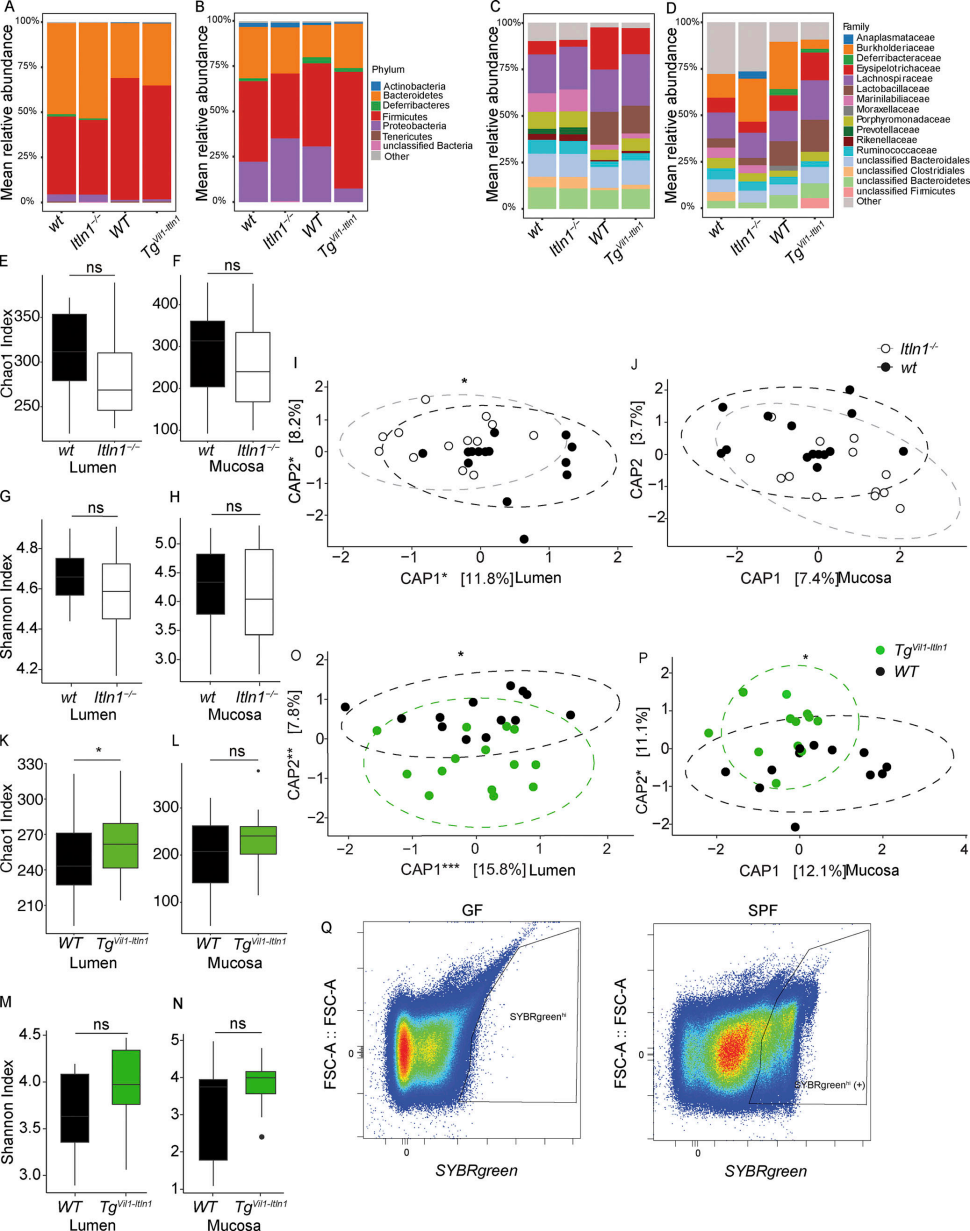
**Baseline characterization of the microbiota of *Itln1***^***−/−***^ **and *Tg***^***Vil-Itln1***^ **mice. (A)** Stacked bars represent the aggregated total community composition at the phyla level in the large intestinal lumen of *Itln1*^*−/−*^ mice and their wild-type littermates (wt; *n* = 14) and *Tg*^*Vil1-Itln1*^ mice and their wild-type littermates (WT; *n* = 13 or 14). **(B)** Stacked bars represent the aggregated total community composition at the phyla level in the colonic mucosa of *Itln1*^*−/−*^ mice and their wild-type littermates (wt; *n* = 14) and *Tg*^*Vil1-Itln1*^ mice compared to wild-type littermates (WT; *n* = 12). **(C)** Stacked bars represent the aggregated total community composition at the family level in the large intestinal lumen of *Itln1*^*−/−*^ mice and their wild-type littermates (wt; *n* = 14) and *Tg*^*Vil1-Itln1*^ mice compared to wild-type littermates (WT; *n* = 13 or 14). **(D)** Stacked bars represent the aggregated total community composition at the family level in the colonic mucosa of *Itln1*^*−/−*^ mice and their wild-type littermates (wt; *n* = 14) and *Tg*^*Vil1-Itln1*^ mice compared to wild-type littermates (WT; *n* = 12). **(E)** Chao1 index for *Itln1*^*−/−*^ mice compared to wild-type littermates (wt) in the large intestinal lumen (*n* = 14). **(F)** Chao1 index for *Itln1*^*−/−*^ mice compared to wild-type littermates (wt) in the colonic mucosa (*n* = 14). **(G)** Shannon index for *Itln1*^*−/−*^ mice compared to wild-type littermates (wt) in the large intestinal lumen (*n* = 14). **(H)** Shannon index for *Itln1*^*−/−*^ mice compared to wild-type littermates (wt) in the colonic mucosa (*n* = 14). **(I)** Beta diversity of the microbiota composition of the large intestinal lumen by Bray–Curtis dissimilarity in *Itln1*^*−/−*^ mice compared to wild-type littermates (wt; *n* = 14). Symbols represent individual mice. **(J)** Beta diversity of the microbiota composition of the colonic mucosa by Bray–Curtis dissimilarity in *Itln1*^*−/−*^ mice compared to wild-type littermates (wt; *n* = 14). Symbols represent individual mice. **(K)** Chao1 index for *Tg*^*Vil1-Itln1*^ mice compared to wild-type littermates (WT) in the large intestinal lumen (*n* = 13 or 14). **(L)** Chao1 index for *Tg*^*Vil1-Itln1*^ mice compared to wild-type littermates (WT) in the colonic mucosa (*n* = 12). **(M)** Shannon index for *Tg*^*Vil1-Itln1*^ mice compared to wild-type littermates (WT) in the large intestinal lumen (*n* = 13 or 14). **(N)** Shannon index for *Tg*^*Vil1-Itln1*^ mice compared to wild-type littermates (WT) in the colonic mucosa (*n* = 12). **(O)** Beta diversity of the microbiota composition of the large intestinal lumen by Bray–Curtis dissimilarity in *Tg*^*Vil1-Itln1*^ mice compared to wild-type littermates (WT; *n* = 13 or 14). Symbols represent individual mice. **(P)** Beta diversity of the microbiota composition of the colonic mucosa by Bray–Curtis dissimilarity in *Tg*^*Vil1-Itln1*^ mice compared to wild-type littermates (WT; *n* = 12). Symbols represent individual mice. **(Q)** Bacteria gating strategy for ITLN1-seq. Stool bacteria were identified as SYBRgreen high (SYBRgreen^hi^) particles in SPF mice that were not present in GF mice. In the SYBRgreen^hi^ fraction, we quantified the fraction of ITLN1(+) bacteria identified with a PE-conjugated ITLN1 antibody, as depicted in Fig. 2 B. P values for the alpha diversity (Chao1 and Shannon index) were calculated using a linear mixed model by regressing the alpha diversity value against the “genotype” with gender as a fixed effect and litter as a random effect (ns: P > 0.05). CAP1 and CAP2 are the first two axes from the constrained analysis of principal coordinates with the respective amount of variation in Bray–Curtis dissimilarity explained between brackets. For Bray–Curtis dissimilarity, P obtained by the anova.cca test with respect to the genotype with 10,000 permutations (*P < 0.05). WT = wild-type littermate from *Tg*^*Vil1-Itln1*^ colony. wt = wild-type littermate from *Itln1*^*−/−*^ colony.

Although ITLN1 expression is limited to Paneth cells in mice (Fig. 2 A, middle panels, arrows), ITLN1 and ITLN2 are expressed in goblet cells and Paneth cells, respectively, in humans (Parikh et al., 2019; Kinchen et al., 2018; Smillie et al., 2019; Martin et al., 2019; Wang et al., 2020; Haber et al., 2017; Almalki et al., 2021; Nonnecke et al., 2021; Nonnecke et al., 2022). We engineered novel mouse lines using the C57BL/6 strain that possesses a single *Itln1* gene unlike several other inbred mouse strains, where duplication of the *Itln1* gene results in up to six paralogs (*Itln1-6*) with variable patterns of intestinal expression (Lu et al., 2011; Tang et al., 2010; Almalki et al., 2021). We generated an *Itln1* knockout line (*Itln1*^*−/−*^) and a transgenic mouse line where ITLN1 expression is driven by the Villin-1 promoter (Pinto et al., 1999; *Tg*^*Vil1-Itln1*^). *Tg*^*Vil1-Itln1*^ mice exhibit IEC-specific overexpression of ITLN1, including colonic goblet cells, thus recapitulating the expression of ITLN1 observed in human colonic intestinal epithelium and especially in human UC when ITLN1 is upregulated (Fig. 2 A). Neither *Itln1*^*−/−*^ nor *Tg*^*Vil1-Itln1*^ mice had differences in abundances of colonic epithelial cell types or lamina propria (LP) leukocyte subtypes at baseline compared with wild-type littermates (Fig. S1, F–G and Fig. S2). ITLN1 is secreted into the intestinal lumen, where it could plausibly interact with and impact the microbiota (Wrackmeyer et al., 2006). We characterized differences in the luminal and mucosa-associated microbial communities between our mice by 16S rRNA gene sequencing. We first examined the composition at the level of the major phyla and families. We found no significant differences between *Itln1*^*−/−*^ and *Tg*^*Vil1-Itln1*^ and their wild-type littermates (Fig. S3, A–D). Next, we examined diversity within communities (alpha diversity) at the level of both species’ richness (Chao1 index) and evenness (Shannon index). This revealed significantly greater lumen microbial richness in *Tg*^*Vil1-Itln1*^ compared with their wild-type littermates, but no differences in any other comparison (Fig. S3, E–H and K–N). Using analyses based on Bray–Curtis dissimilarity to evaluate overall differences between communities (beta diversity), we detected significant differences in luminal communities when comparing *Itln1*^−/−^ and *Tg*^*Vil1-Itln1*^ mice with their respective wild-type littermates (Fig. S3, I and O). Colonic mucosa-associated bacteria also differed between *Tg*^*Vil1-Itln1*^ and wild-type littermates (Fig. S3 P), but not between *Itln1*^−/−^ and wild-type littermates (Fig. S3 J). No amplicon sequence variants (ASVs) were significantly enriched in the mucosa among the different mouse lines. However, a few taxa differed in the luminal communities of *Tg*^*Vil1-Itln1*^ mice compared with their wild-type littermates (Table S1). Thus, despite minor differences, there are no prominent effects of *Itln1* knockout or overexpression on global intestinal microbial community composition.

### ITLN1 binds a select group of microorganisms in vivo, including *A. muciniphila*

To further investigate whether ITLN1 interacts with specific bacterial species within the gut microbiota in vivo, we developed a flow cytometry–based assay to analyze in vivo ITLN1 binding to commensal bacteria in *Tg*^*Vil1-Itln1*^ mice or their wild-type littermates using *Itln1*^−/−^ mice as a negative control. Similar assays have been used to study IgA and surfactant protein D–bound organisms (Palm et al., 2014; Sarashina-Kida et al., 2017). ITLN1 bound a small subset (∼1%) of microbiota in *Tg*^*Vil1-Itln1*^ mice, a nearly twofold increase over wild-type littermates, where only ∼0.5% of bacteria were ITLN1-positive (Fig. 2 B and Fig. S3 Q).

**Figure S4.**
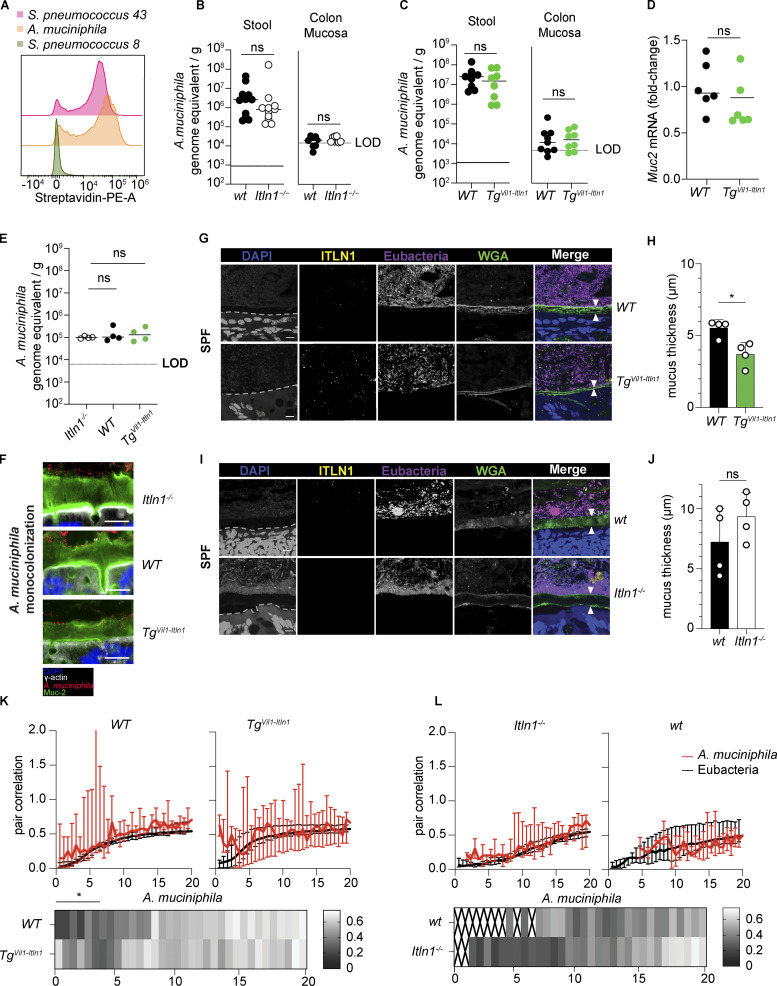
***A muciniphila* binding to ITLN1 in vitro, *A. muciniphila* quantification by qPCR, and methacarn and methacrylate fixed tissue imaging. (A)** Representative histogram showing binding of recombinant ITLN1 to *A. muciniphila* isolated from *Tg*^*Vil-Itln1*^ mice in orange, negative control in green (*Streptococcus pneumoniae* serotype 8), and positive control in red (*Streptococcus pneumoniae* serotype 43; *n* = 9). **(B)** Absolute abundance of *A. muciniphila* in *Itln1*^*−/−*^ mice compared to wild-type littermates by qPCR in stools and colon mucosa (*n* = 8–11). Symbols represent individual mice. Bars represent arithmetic means. Dotted lines represent the level of detection (LOD). **(C)** Absolute abundance of *A. muciniphila* in *Tg*^*Vil1-Itln1*^ mice compared to wild-type littermates by qPCR in stools and colon mucosa (*n* = 8). Symbols represent individual mice. Bars represent arithmetic means. Dotted lines represent the LOD. **(D)** Quantification of *Muc2* transcripts by qPCR in colonic biopsies of WT and *Tg*^*Vil1-Itln1*^ mice (*n* = 6). Symbols represent individual mice. Bars represent arithmetic means. **(E)** Absolute abundance of *A. muciniphila* in wt, *Itln1*^*−/−*^, and *Tg*^*Vil1-Itln1*^ mice in stools of ex-GF mice monocolonized by *A. muciniphila* compared to wild-type littermates by qPCR from Fig. 3, H and I (*n* = 4). Symbols represent individual mice. Bars represent arithmetic means. **(F)** Representative fluorescent images obtained after methacarn fixation and combined immunofluorescence (IF) and FISH showing *A. muciniphila* signal in the colonic inner mucus layer of ex-GF monocolonized *Itln1*^*−/−*^, WT, and *Tg*^*Vil1-Itln1*^ mice from Fig. 3, H and I (*n* = 4). For each genotype, we present representative fluorescence merged images with pseudocoloring depicting the nuclei (DAPI = blue), the intestinal epithelial surface (γ-actin IF = gray), *A. muciniphila* (*A. muciniphila* probe FISH = *A. muciniphila* = red), and the mucus layer (Mucin-2 IF = MUC2 = green). **(G)** Representative fluorescence images obtained after methacrylate fixation and combined IF and FISH of distal colon from *Tg*^*Vil1-Itln1*^ mice and their respective wild-type littermates (WT) under SPF conditions. For each genotype, we present representative grayscale images depicting the nuclei and epithelial cell autofluorescence (DAPI), ITLN1, intestinal microbiota (Eubacteria probes FISH = Eubacteria), and the mucus layer (FITC labeled WGA) and merged image with pseudo coloring (DAPI = blue, ITLN1 = yellow, Eubacteria = magenta, WGA = green). The inner mucus layer was characterized by a well-organized stratified WGA lamellar appearance between white arrowheads in the merged image. The dashed line represents the apical epithelial edge identified by DAPI autofluorescence. Scale bars indicate 10 µm. **(H)** Inner mucus thickness was measured between white arrowheads in G as described by Earle et al. (2015). 143–144 independent measurements 50 μm apart were obtained from four different mice per genotype (*n* = 4). Mean and SD were determined from the average measurements for each mouse. Each dot represents the mean value of measurements per mouse. Error bars represent the SD. **(I)** Representative fluorescence images obtained after methacrylate fixation and combined IF and FISH of distal colon from *Itln1*^*−/−*^ mice and their respective wild-type littermates (wt) under SPF conditions. For each genotype, we present representative grayscale images depicting the nuclei and epithelial cell autofluorescence (DAPI), ITLN1, intestinal microbiota (Eubacteria probes FISH = Eubacteria), and the mucus layer (FITC labeled WGA) and merged image with pseudo coloring (DAPI = blue, ITLN1 = yellow, Eubacteria = magenta, WGA = green). The inner mucus layer was characterized by a well-organized stratified WGA lamellar appearance between white arrowheads in the merged image. The dashed line represents the apical epithelial edge identified by DAPI autofluorescence. Scale bars indicate 10 µm. **(J)** Inner mucus thickness was measured between white arrowheads in I as described by Earle et al. (2015). 144 independent measurements 50 μm apart were obtained from four different mice per genotype (*n* = 4). Mean and SD were determined from the average measurements for each mouse. Each dot represents the mean value of measurements per mouse. Error bars represent the SD. **(K)** Proximity analysis of the overall bacterial distribution (Eubacteria) and distribution of *A. muciniphila* in the inner mucus layer in methacrylate fixed tissues (*n* = 4). Distances relative to the epithelial-facing edge of the mucus layer. Higher/lower pair correlation values indicate a relative attraction/repulsion to the mucus edge close to the epithelium. The average pair correlation value for each distance point was calculated from average measurements for each mouse (3 fields of view/section × 3 sections/animal × 4 animals/genotype = 36 fields of view per genotype). The error bar for each distance point represents the 95% confidence interval. **(L)** Proximity analysis of the overall bacterial distribution (Eubacteria) and distribution of *A. muciniphila* with respect to the mucus layer in methacrylate fixed tissues. Distances relative to the epithelial-facing edge of the mucus layer. Higher/lower pair correlation values indicate a relative attraction/repulsion to the mucus edge close to the epithelium. The average pair correlation value for each distance point was calculated from average measurements for each mouse (3 fields of view/section × 3 sections/animal × 4 animals/genotype = 36 fields of view per genotype). The error bar for each distance point represents the 95% confidence interval. WT = wild-type littermate from *Tg*^*Vil1-Itln1*^ colony. wt = wild-type littermate from *Itln1*^*−/−*^ colony. P values were calculated by unpaired *T* test (B–D, H, J, and K) or one-way ANOVA with Dunnet (E). *P < 0.05.

To identify the specific bacterial taxa bound to ITLN1, we sorted ITLN1-positive and ITLN1-negative fecal bacteria from *Tg*^*Vil1-Itln1*^ mice and performed 16S rRNA gene sequencing (ITLN1-seq). Comparison using Bray–Curtis dissimilarity demonstrated a statistically significant difference (P = 10^−4^ by PERMANOVA) between these fractions (Fig. 2 C), suggesting that ITLN1 binds to a highly restricted group of bacteria. Phylogenetic analysis revealed that the order *Verrucomicrobiales* was significantly enriched in the ITLN1-positive fraction compared with the ITLN1-negative fraction (Fig. 2 D). *Verrucomicrobiales* is an order of commensals in the Verrucomicrobia phylum, which until recently only contained one member, *A. muciniphila* (Derrien et al., 2004; Ansaldo et al., 2019). Consistent with the in vivo mouse data, human recombinant ITLN1 bound *A. muciniphila* isolated from human samples and cultured ex vivo (Derrien et al., 2004; Fig. 2 E) at quantitatively comparable levels to the known ITLN1-binding bacterial strain *Streptococcus pneumoniae* serotype 43 (Wesener et al., 2015; Fig. 2 E). Results were similar when *A. muciniphila* from *Tg*^*Vil1-Itln1*^ mice was analyzed (Fig. S4 A). These data suggest that the glycan moiety containing terminal exocyclic 1,2-diols that mediates interactions between ITLN1 and bacteria is present in *A. muciniphila* isolates from human subjects and mice. Despite this, the abundance of fecal or colonic mucosa-associated *A. muciniphila* was similar across the different ITLN1 mouse models (Fig. S4, B and C). Thus, although *A. muciniphila* is a prominent member of the small subset of gut microbes bound by ITLN1, its abundance does not appear to be regulated by ITLN1.

### ITLN1 facilitates thinning of the inner colonic mucus layer in a microbiota-dependent manner

**Figure 3.**
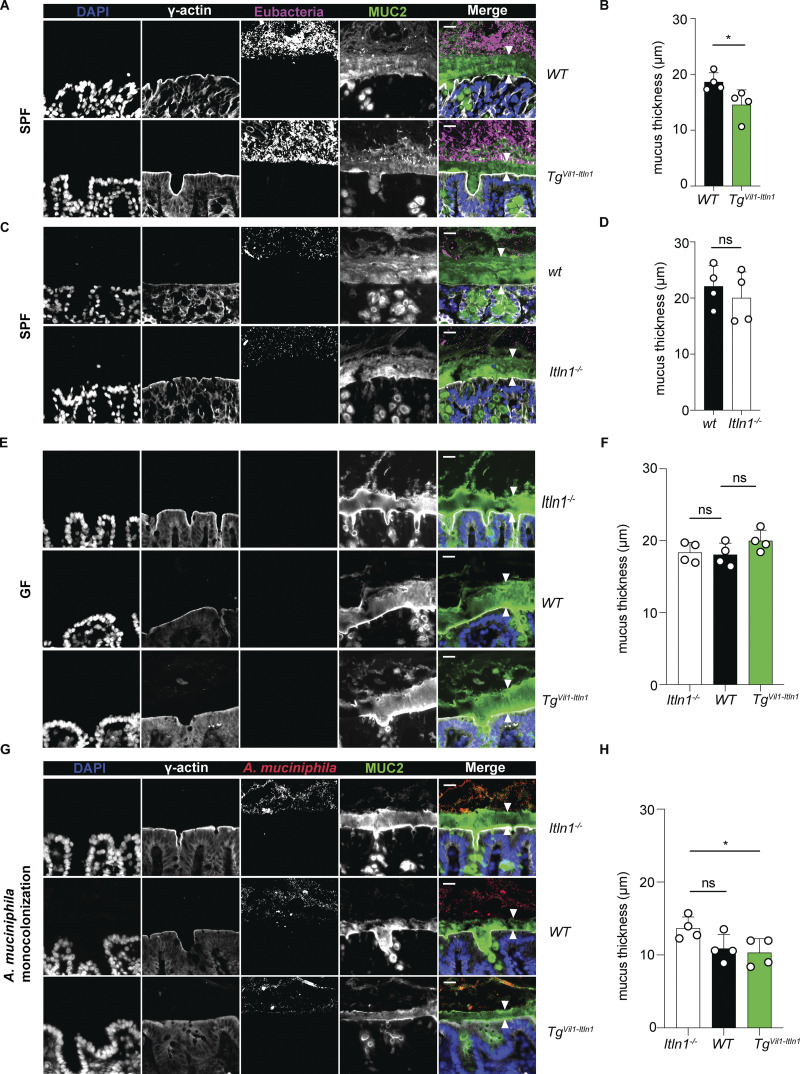
**ITLN1 affects the inner mucus layer thickness in a microbiota-dependent manner. (A)** Representative fluorescence images obtained after methacarn fixation and combined immunofluorescence (IF) and FISH of the distal colon from *Tg*^*Vil1-Itln1*^ mice and their respective wild-type littermates (WT) under SPF conditions. For each genotype, we present representative grayscale images depicting the nuclei (DAPI), the intestinal epithelial surface (γ-actin IF = γ-actin), the intestinal microbiota (Eubacteria probes FISH = Eubacteria), and the mucus layer (Mucin-2 IF = MUC2) and merged image with pseudo coloring (DAPI = blue, γ-actin = gray, Eubacteria = magenta, MUC2 = green). The inner mucus layer was characterized by a well-organized stratified MUC2 lamellar appearance between the epithelium and the bacterial biomass in the merged image (between white arrowheads). Scale bars indicate 20 µm. **(B)** Inner mucus thickness was measured between white arrowheads in A as described by Earle et al. (2015). 288 independent measurements 50 μm apart were obtained from four different mice per genotype (*n* = 4). Mean and SD were determined from the average measurements for each mouse. Each dot represents the mean value of measurements per mouse. Error bars represent the SD. **(C)** Representative fluorescence images were obtained after methacarn fixation and combined IF and FISH of distal colon from *Itln1*^*−/−*^ mice and their respective wild-type littermates (wt) under SPF conditions. For each genotype, we present representative fluorescence images depicting the nuclei (DAPI), the intestinal epithelial surface (γ-actin IF = γ-actin), intestinal microbiota (Eubacteria probes FISH = Eubacteria), and the mucus layer (Mucin-2 IF = MUC2) and merged images with pseudo coloring (DAPI = blue, γ-actin = gray, Eubacteria = magenta, MUC2 = green). The inner mucus layer was characterized by a well-organized stratified MUC2 lamellar appearance between the epithelium and the bacterial biomass in the merged image (between white arrowheads). Scale bars indicate 20 µm. **(D)** Inner mucus thickness was measured between white arrowheads in C as described by Earle et al. (2015). 276–284 independent measurements 50 μm apart were obtained from four different mice per genotype (*n* = 4). Mean and SD were determined from the average measurements for each mouse. Each dot represents the mean value of measurements per mouse. Error bars represent the SD. **(E)** Representative fluorescence images obtained after methacarn fixation and combined IF and FISH of distal colon from GF wild-type, *Itln1*^*−/−*^, and *Tg*^*Vil1-Itln1*^ mice. For each genotype, we present representative fluorescence images depicting the nuclei (DAPI), the intestinal epithelial surface (γ-actin IF = γ-actin), intestinal microbiota (Eubacteria probes FISH = Eubacteria), and the mucus layer (Mucin-2 IF = MUC2) and merged images with pseudo coloring (DAPI = blue, γ-actin = gray, Eubacteria = magenta, MUC2 = green). The inner mucus layer was characterized by a well-organized stratified MUC2 lamellar appearance above the epithelium (between white arrowheads). Scale bars indicate 20 µm. **(F)** Inner mucus thickness was measured between white arrowheads in E as described by Earle et al. (2015). 244–276 independent measurements 50 μm apart were obtained from four different mice per genotype (*n* = 4). Mean and SD were determined from the average measurements for each mouse. Each dot represents the mean value of measurements per mouse. Error bars represent the SD. **(G)** Representative fluorescence images obtained after methacarn fixation and combined IF and FISH of distal colon from GF wild-type, *Itln1*^*−/−*^, and *Tg*^*Vil1-Itln1*^ mice monocolonized with *A. muciniphila*. For each genotype, we present representative fluorescence images depicting the nuclei (DAPI), the intestinal epithelial surface (γ-actin IF = γ-actin), *A. muciniphila* (*A. muciniphila* probe FISH = *A. muciniphila*), and the mucus layer (Mucin-2 IF = MUC2) and merged images with pseudo coloring (DAPI = blue, γ-actin = gray, *A. muciniphila* = red, mucus layer = green). The inner mucus layer was characterized by a well-organized stratified MUC2 lamellar appearance between the epithelium and the bacterial biomass in the merged image (between white arrowheads). Scale bars indicate 20 µm. **(H)** Inner mucus thickness was measured between white arrowheads in G as described by Earle et al. (2015). 244–276 independent measurements 50 μm apart were obtained from four different mice per genotype (*n* = 4). Mean and SD were determined from the average measurements for each mouse. Each dot represents the mean value of measurements per mouse. Error bars represent the SD. WT = wild-type littermate from *Tg*^*Vil1-Itln1*^ colony. wt = wild-type littermate from *Itln1*^*−/−*^ colony. P values were calculated by unpaired *T* test (B and D) and one-way ANOVA corrected for multiple comparisons with Dunnet (F and H). *P < 0.05.

ITLN1 is part of the core mucus proteome, and attenuation of the colonic mucus layer is an early event in UC (van der Post et al., 2019). Altered *A. muciniphila* abundance has been associated with changes in the mucus layer in mice (Li et al., 2017; Bian et al., 2019; Seregin et al., 2017; Ganesh et al., 2013; Zhang et al., 2021; Kim et al., 2021). Given *A. muciniphila’*s known mucolytic properties (Derrien et al., 2004) and its binding to ITLN1, we hypothesized that ITLN1, in combination with *A. muciniphila*, might affect the thickness of the colonic mucus layer. We evaluated the attached inner mucus layer in distal colon samples using methanol-Carnoy (methacarn) fixation, as previously reported (Desai et al., 2016; Earle et al., 2015; Johansson and Hansson, 2011; Welch et al., 2017; Musch et al., 2013; Jakobsson et al., 2015; Bergstrom et al., 2020). Under SPF conditions, the inner mucus layer was characterized by a well-organized stratified mucin-2 (MUC2) lamellar appearance, delimitated by the microbiota on the luminal side (Johansson et al., 2011; Johansson et al., 2015), and γ-actin, a marker of the apical epithelial cell lining (Kaji et al., 2020). Overexpression of ITLN1 was associated with a statistically significant reduction in the thickness of the colonic inner mucus layer (18.63 mm with an SD of ±1.66 μm in WT mice versus 14.55 ±2.68 μm in *Tg*^*Vil1-Itln1*^; Fig. 3, A and B), whereas the measurement of the colonic inner mucus layer was not significantly different in *Itln1*^*−/−*^ mice relative to their wild-type littermates (22.08 ± 3.69 μm in wt mice versus 20.01 ± 4.65 mm in *Itln1*^*−/−*^; Fig. 3, C and D). Despite reports that *A. muciniphila* has paracrine proliferative effects on goblet cells (Kim et al., 2021), colonic goblet cell numbers (Fig. S1 G) and *Muc2* expression were similar in *Tg*^*Vil1-Itln1*^ mice and their wild-type littermates (Fig. S4 D). This may reflect the absence of changes in overall *A. muciniphila* abundance. To determine whether microbiota contributed to the observed phenotype, we rederived *Tg*^*Vil1-Itln1*^ and *Itln1*^*−/−*^ under GF conditions. The colonic inner mucus layer thickness was similar in GF wild-type, *Tg*^*Vil1-Itln1*^, and *Itln1*^*−/−*^ mice (Fig. 3, E and F). These results suggest that the differences observed under SPF conditions in the inner mucus layer thickness between *Tg*^*Vil1-Itln1*^ mice and their wild-type littermates are microbiota dependent. To further test our hypothesis that *A. muciniphila* is critical to the reduced mucus thickness of SPF *Tg*^*Vil1-Itln1*^ mice, we monocolonized GF mice with *A. muciniphila*. Within 3 wk of monocolonization, the inner mucus layer of *Tg*^*Vil1-Itln1*^ mice was significantly thinner than that of *Itln1*^*−/−*^ mice. There was also a trend toward reduced inner mucus layer thickness in monocolonized wild-type mice relative to *Itln1*^−/−^ mice (Fig. 3, G and H). Nevertheless, fecal *A. muciniphila* content was similar across genotypes (Fig. S4 E), suggesting that the difference observed in the mucus layer thicknesses was not related to total *A. muciniphila* biomass but depends on the copresence of *A. muciniphila* and ITLN1. Furthermore, we did note that ITLN1 expression, in both wild-type and *Tg*^*Vil1-Itln1*^ mice, was associated with increased *A. muciniphila* infiltration of the stratified mucus layer relative to *Itln1*^*−/−*^ mice (Fig. S4 F). To better assess microbial communities morphologically under SPF conditions, tissues were fixed in methacrylate, which preserves the three-dimensional structure of the intestinal microbiota (Welch et al., 2017; Hasegawa et al., 2017). As expected, the inner mucus layer, defined as the wheat germ agglutinin (WGA) stratified layer between the bacterial biomass and the epithelial border, of *Tg*^*Vil1-Itln1*^ mice was thinner than that of wild-type littermates in methacrylate-fixed tissues (Fig. S4, G–H). Also, consistent with results using methacarn-fixed tissues, there were no differences between the inner mucus layer thickness of *Itln1*^*−/−*^ mice and their wild-type littermates in methacrylate-fixed tissues (Fig. S4, I and J). Despite this, absolute measurements of inner mucus layer thickness differed between tissues fixed in methacarn or methacrylate, likely related to differences in fixative composition and, possibly, that the tissues were not harvested and fixed in parallel (Earle et al., 2015). Nevertheless, the results using methacrylate or methacarn fixation consistently show that under SPF conditions the inner mucus layer of *Tg*^*Vil1Itln1*^ is thinner than that of their wild-type littermates.

Having validated methacrylate fixation, we conducted proximity analyses to quantify if the ITLN1-associated reduction in mucus thickness affected bacterial penetration of the inner mucus layer under SPF conditions. As previously reported, the bulk of the bacteria (Eubacteria in Fig. S4 K) displayed a repulsive relationship toward the mucus edge close to the epithelium (Welch et al., 2017; Hasegawa et al., 2017; Johansson et al., 2010; Vaishnava et al., 2011). However, species-specific proximity measurements revealed that *A. muciniphila* localized significantly closer to the epithelial surface (<3 μm) in *Tg*^*Vil1-Itln1*^ mice (Fig. S4 K) compared to their wild-type littermates. Signals close to the epithelium using the fluorescent in situ hybridization (FISH) probe against *A. muciniphila* were scarce under SPF conditions in *Itln1*^*−/−*^ and wild-type littermates. Therefore, meaningful comparisons were not possible (Fig. S4 L). Together, these findings suggest that ITLN1 facilitates colonization of inner colonic mucus by *A. muciniphila* and mucus thinning in support of the hypothesis that mucus thinning is secondary to the mucolytic activity of *A. muciniphila*. We hypothesize that the lack of any differences in the inner mucus layer thickness between wild-type and *Itln1*^*−/−*^ mice under SPF conditions, compared with the trend observed in mice monocolonized with *A. muciniphila,* may reflect unidentified microbial influences that protect the inner colonic mucus layer and can only be overcome by overexpression of ITLN1 under SPF conditions.

### ITLN1 overexpression is associated with increased intestinal inflammation

**Figure S5.**
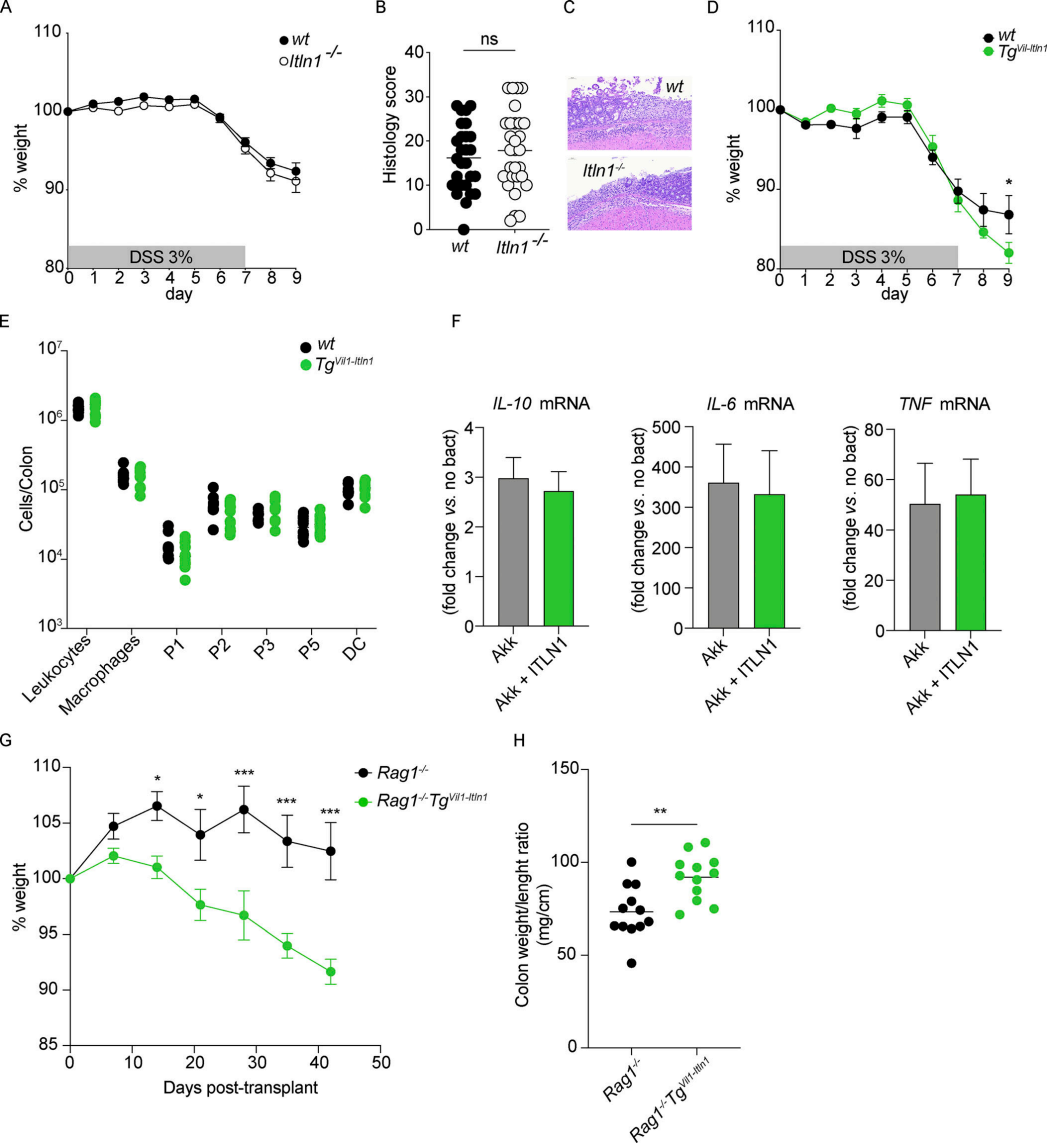
**ITLN1 over expression worsens colitis in DSS-induced and naive T transfer-induced colitis model. (A)** Weight loss after exposure to DSS for 7 and 2 d of water (DSS colitis) in *Itln1*^*−/−*^ mice and wild-type littermates (WT; *n* = 34). Symbols represent means of baseline weight. Error bars represent SEs. Data were compiled from three independent experiments. **(B)** Histology score on day 8 or 9 following DSS colitis (*n* = 27 or 30). Symbols represent individual mice. Data were compiled from three independent experiments. **(C)** Representative micrograph of *Itln1*^*−/−*^ and wt mice after DSS colitis. **(D)** Weight loss after DSS colitis in *Tg*^*Vil1-Itln1*^ mice and wild-type littermates (WT; *n* = 10–11) in a separate cohort of mice treated with DSS for the cytokines explant experiment in Fig. 4, D and E. Symbols represent means of baseline weight. Error bars represent SEs. Data were compiled from two independent experiments. **(E)** Quantification of macrophages in colonic LP from *Tg*^*Vil1-Itln1*^ mice compared to wild-type littermates during day 2 of DSS colitis (*n* = 10–11). **(F)** Transcription of *IL-10*, *IL-6*, and *TNF* in human monocyte-derived macrophages following incubation with uncoated (Akk) or ITLN1-coated *A. muciniphila* (Akk + ITLN1; *n* = 3–4 from two independent experiments). **(G)** Weight loss after transfer of naive T cells in *Tg*^*Vil1-Itln1*^ *Rag1*^*−/−*^ and *Rag1*^*−/−*^ littermates (*n* = 11). Symbols represent means of baseline weight. Error bars represent SEs. **(H)** Colonic weight to length ratio (Ostanin et al., 2008) 6 wk after transfer of naive T cells in *Tg*^*Vil1-Itln1*^ *Rag1*^*−/−*^ and *Rag1*^*−/−*^ littermates (*n* = 11). Symbols represent individual mice and bars represent the means. P values were calculated by unpaired *T* test with correction for multiple comparisons using Holm–Sidak in (A, D, and G) and unpaired *T* test in (B, E, F, and H). *P < 0.05; **P < 0.01; ***P < 0.001.

**Figure 4.**
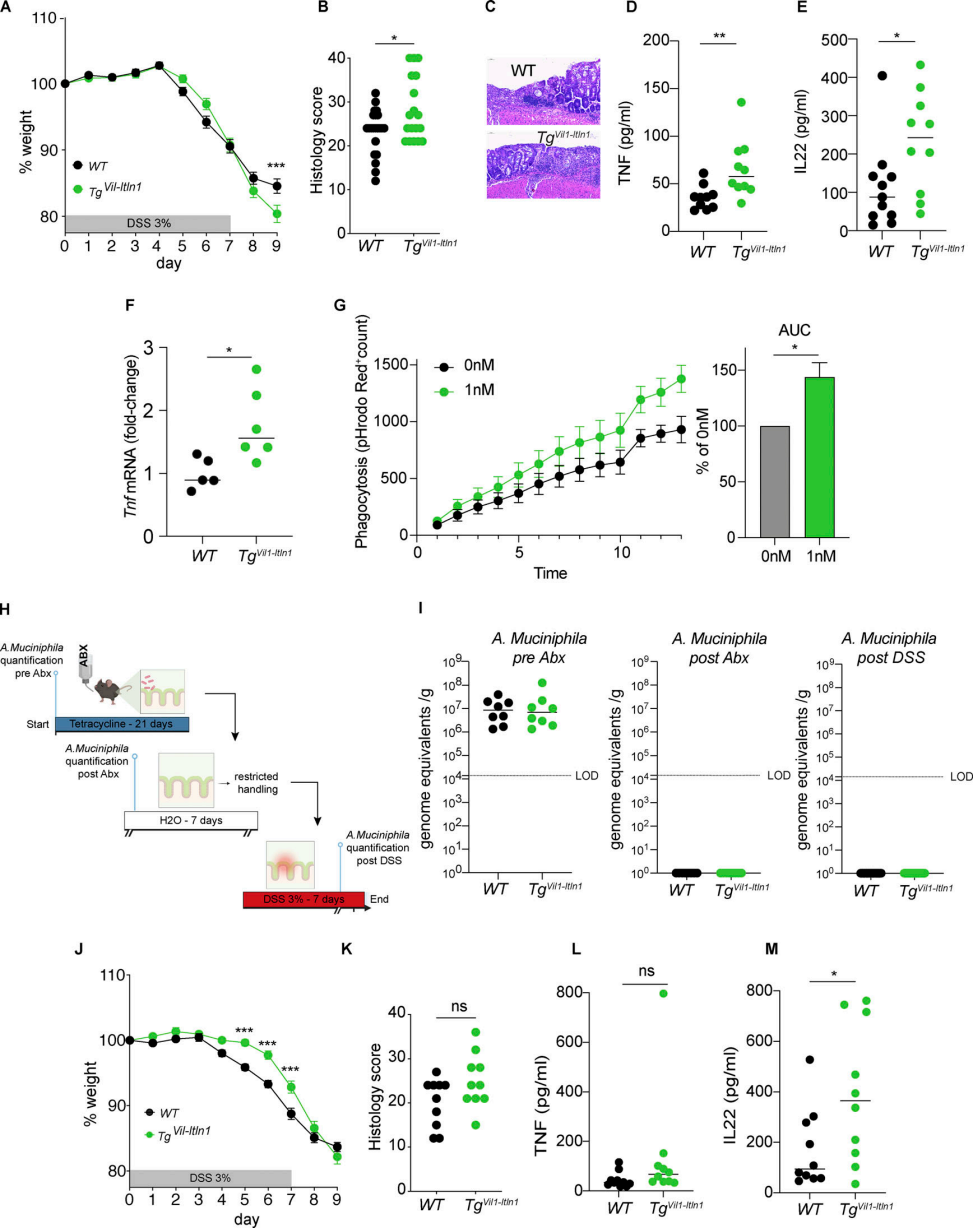
**Overexpression of ITLN1 results in increased susceptibility to colitis ameliorated after clearance of *A. muciniphila* with tetracycline. (A)** Weight loss after exposure to DSS for 7 and 2 d of water (DSS colitis) in *Tg*^*Vil1-Itln1*^ and wild-type littermates (WT; *n* = 19 or 22). Symbols represent means of baseline weight. Error bars represent SEs. Data were compiled from three independent experiments. **(B)** Histology score on day 9 following DSS colitis (*n* = 19 or 22). Symbols represent individual mice. Data were compiled from three independent experiments. **(C)** Representative micrograph of *Tg*^*Vil1-Itln1*^ and wild-type littermates (WT) after DSS colitis on day 9. **(D)** TNF measurement on colonic explants from *Tg*^*Vil1-Itln1*^ and wild-type littermates (WT) on day 9 after DSS colitis (*n* = 10). Symbols represent individual mice. Data were compiled from two independent experiments (see weight loss graph in Fig. S5 D). **(E)** IL-22 measurement on mouse colonic explants after DSS colitis (*n* = 10–11). Symbols represent individual mice. Data were compiled from two independent experiments (see weight loss graph in Fig. S5 D). **(F)** *Tnf* transcription in sorted macrophages from *Tg*^*Vil1-Itln1*^ and wild-type littermates (WT) during day 2 of DSS colitis (*n* = 5–6). **(G)** Phagocytosis of uncoated (0 nM) or ITLN1-coated (1 nM) pHrodo Red–conjugated *A. muciniphila* by human monocyte-derived macrophages. The left panel shows the signal increase over time, and the right panel shows the corresponding AUC. *n* = 4. Data were compiled from two independent experiments. **(H)** Schematic of tetracycline treatment for 3 wk to eradicate *A. muciniphila*. **(I)** Absolute *A. muciniphila* levels by qPCR in stools before treatment with tetracycline (left panel), after treatment with tetracycline (middle panel), and after DSS experiment (right panel) in *Tg*^*Vil1-Itln1*^ and wild-type littermates (WT; *n* = 8–19). Symbols represent individual mice. **(J)** Weight loss after DSS colitis model in *Tg*^*Vil1-Itln1*^ and wild-type littermates (WT) treated with tetracycline (*n* = 20). Symbols represent means of baseline weight. Error bars represent SEs. Data were compiled from three independent experiments. **(K)** Histology score on day 9 following DSS colitis after tetracycline treatment in *Tg*^*Vil1-Itln1*^ and wild-type littermates (WT; *n* = 10). Symbols represent individual mice. **(L)** TNF measurement on colonic explants from *Tg*^*Vil1-Itln1*^ and wild-type littermates (WT) on day 9 after DSS colitis following tetracycline treatment (*n* = 10). Symbols represent individual mice. **(M)** IL22 measurement on colonic explants from *Tg*^*Vil1-Itln1*^ and wild-type littermates (WT) on day 9 after DSS colitis following tetracycline treatment (*n* = 10). Symbols represent individual mice. WT = wild-type littermate from *Tg*^*Vil1-Itln1*^ colony. wt = wild-type littermate from *Itln1*^*−/−*^ colony. P values were calculated by unpaired *T* test with correction for multiple comparisons using Holm–Sidak (A and J) or unpaired *T* test (B, D–G, I, and K–M). *P < 0.05; **P < 0.01; ***P < 0.001.

*Muc2*-deficient mice have an impaired mucus layer and are highly susceptible to spontaneous and chemically induced (i.e., DSS) colitis (Van der Sluis et al., 2006). *A. muciniphila* has additionally been associated with a decreased mucus layer and spontaneous colitis in GF *Il10*^*−/−*^ mice (Seregin et al., 2017). *Il10*^−/−^ mice exhibit intestinal epithelial ER stress (Shkoda et al., 2007). Since *Tg*^*Vil1-Itln1*^ mice exhibit decreased inner mucus layer thickness but did not differ from wild-type littermates in *A. muciniphila* abundance, we tested the susceptibility of *Tg*^*Vil1-Itln1*^ and *Itln1*^*−/−*^ mice to DSS-induced colitis. Colitis severity was similar in SPF *Itln1*^−/−^ mice colitis and wild-type littermates (Fig. S5, A–C). In contrast, *Tg*^*Vil1-Itln1*^ mice exhibited more severe weight loss and histologic damage than wild-type littermates after DSS administration (Fig. 4, A–C; and Fig. S5 D). Consistent with this, intestinal explants of DSS-treated *Tg*^*Vil1-Itln1*^ mice produced more TNF (Nava et al., 2010; Fig. 4 D) and IL-22 (Sugimoto et al., 2008; Fig. 4 E) than explants of DSS-treated wild-type littermates. TNF is mainly produced by macrophages during DSS colitis (Jones et al., 2018). We, therefore, compared mucosal macrophage populations of wild-type and *Tg*^*Vil1-Itln1*^ mice after the DSS challenge. Although we did not detect differences in the number or percentage of monocytes or macrophages in the intestine (Tamoutounour et al., 2012) during DSS colitis on day 2 (Fig. S5 E), in vivo *Tnf* transcription by LP macrophages of *Tg*^*Vil1-Itln1*^ mice was greater than that of wild-type littermates (Fig. 4 F). One possible explanation for the difference in macrophage activation could be due to differences in bacterial phagocytosis as ITLN1 has been shown to promote phagocytosis of Bacillus Calmette–Guérin bacteria (Tsuji et al., 2009). Indeed, coating with ITLN1 in vitro enhanced *A. muciniphila* phagocytosis by primary human macrophages (Fig. 4 G). It did not affect the transcription of *TNF*, *IL-6*, and *IL-10* (Keely et al., 2014), suggesting that additional proinflammatory cues, as provided during DSS colitis, are required to induce TNF upregulation (Fig. S5 F). Therefore, in addition to decreased mucus thickness and increased proximity of luminal microbiota to the IEC surface, our results suggest that, upon barrier disruption, bacterial coating by ITLN1 in *Tg*^*Vil1-Itln1*^ mice may enhance bacterial phagocytosis and TNF production by mucosal macrophages.

To determine whether the changes observed in chemical colitis extend to the pathophysiology of human IBD, we asked if the increased susceptibility to inflammation extended to the T cell model of colitis (Powrie et al., 1994; Raju et al., 2020), where mucus disruption has been associated with inflammation (Brasseit et al., 2016). After transfer of naive T cells, disease, measured as weight loss and increased colonic weight-to-length ratio (Ostanin et al., 2008), was significantly greater in *Tg*^*Vil1-Itln1*^ *Rag1*^*−/−*^ mice relative to *Rag1*^*−/−*^ littermates (Fig. S5, G and H). Therefore, the increased susceptibility to inflammation-associated mucosal damage is not unique to DSS colitis but also occurs in T cell–mediated colitis.

Lastly, to assess the impact of *A. muciniphila* on colitis severity in Tg^*Vil1-Itln1*^ mice, we depleted *A. muciniphila* using tetracycline (Ansaldo et al., 2019; Fig. 4 H). After verifying *A. muciniphila* elimination (Fig. 4 I), mice were subjected to DSS colitis. There was no longer any difference in weight loss between Tg^*Vil1-Itln1*^ and their wild-type littermates at day 9 (Fig. 4 J). In fact, Tg^*Vil1-Itln1*^ mice were significantly resistant to weight loss between days 5 and 7 of DSS colitis upon tetracycline treatment (Fig. 4 J). Similarly, differences in DSS-induced histologic damage were not significantly different in tetracycline-treated mice (Fig. 4 K). Finally, tetracycline treatment eliminated differences in explants’ TNF production (Fig. 4 L), although IL22 differences persisted (Fig. 4 M). As a whole, these results show that *A. muciniphila* clearance under SPF conditions attenuates the DSS phenotype of Tg^*Vil1-Itln1*^ mice.

## Discussion

Despite its genetic association with IBD (Jostins et al., 2012; Ellinghaus et al., 2016; Huang et al., 2017; Liu et al., 2015), known microbial-binding properties (Wesener et al., 2015; McMahon et al., 2020), and evolutionary conservation (Chen et al., 2020), the regulation and function of ITLN1 at the intestinal host–microbiota interface have remained enigmatic. Here, we used a variety of approaches, including human patient datasets and biopsies, in vitro cell line and stem cell models, and novel mouse models under SPF and GF conditions to evaluate ITLN1 expression, regulation, and function at the host–microbiota interface. We show that the UPR upregulates ITLN1 expression in IECs. Moreover, our data demonstrate that ITLN1 targets specific microbes known to have mucolytic activity. Together, these results suggest that ITLN1 modifies colonic inner mucus layer structure and overall susceptibility to intestinal inflammation and injury in concert with targeted microbes.

The IEC-associated UPR has been linked to intestinal inflammation (Kaser et al., 2008; Grootjans et al., 2016; Grootjans et al., 2019; Adolph et al., 2013; Hosomi et al., 2017; Niederreiter et al., 2013; Stengel et al., 2020; Tréton et al., 2011). We now provide evidence that ITLN1 expression correlates with the ER stress in intestinal epithelia of UC patients. We used experimental models to show that two branches of the UPR—the IRE1α-XBP1 and ATF6 pathways (Grootjans et al., 2016)—are instrumental in regulating ITLN1 expression. The PERK pathway might also be involved, but results using mouse organoids and human intestinal epithelial Caco-2 cells were inconsistent. These differences might reflect cell line or species-specific differences that need further study. Future studies will also be of interest to define the precise mechanism by which the multiple ER stress branches regulate ITLN1 expression in humans and mice. For example, it will be important to determine whether *ITLN1* is a direct target of XBP1 or ATF6. Prior evaluation of the human *ITLN1* promotor has shown that the region of −299/+63 of the transcription start site elicits the maximal promoter activity in transfected Caco-2 cells. However, no UPR-responsive elements were identified within this region (Jiang and Lönnerdal, 2018).

Our finding linking ITLN1 to ER stress provides a mechanistic link between these otherwise separate genetically identified IBD risk factors (Jostins et al., 2012; Ellinghaus et al., 2016; Huang et al., 2017; Liu et al., 2015). We also observed in patients with CD increased intelectin expression in small intestine crypts that were GRP78^+^, a marker of ER stress (Deuring et al., 2014). However, the antibody used does not distinguish between ITLN1 and ITLN2. With recent reports that ITLN2 is the principal intelectin expressed in Paneth cells (Nonnecke et al., 2021; Nonnecke et al., 2022; Wang et al., 2020), our data from CD patients raise the possibility that the UPR may also regulate ITLN2 expression. Other *Itln* genes detected in non-C57BL/6 mice seem to be orthologs of *Itln1*. In contrast, no ortholog of human *ITLN2* has been identified in mice (Almalki et al., 2021; Nonnecke et al., 2022). Further studies involving human tissues, human-derived organoids, or humanized mice will therefore be required to define the parallels and differences between ITLN1 and ITLN2, including their regulation by the UPR. A recent study (Nonnecke et al., 2021) showed increased ITLN1 in colonic patients with UC. It concluded that IBD-associated Single nucleotide polymorphisms (SNPs) close to the *ITLN1* locus do not regulate *ITLN1* expression. It remains to be determined whether any SNPs in linkage disequilibrium with *ITLN1* and *ITLN2* might regulate their expression in the context of ER stress and the UPR. Alternatively, environmental effects, including the inflammatory response per se, may modulate the UPR-induced *ITLN1* and *ITLN2* expression in IBD patients. Together, these observations suggest that dysregulated ITLN1 expression, by either genetic or environmental mechanisms, might influence the development of IBD.

An essential aspect of our studies was the development of novel mouse models that allow in vivo analysis of ITLN1 function. By generating the *Itln1* knockout model (*Itln1*^*−/−*^) using the C57BL/6 strain, we developed a bona fide ITLN1 loss of function model. We overcame the complexity of biologic interpretation introduced by duplication of *Itln1* in other mouse strains (Lu et al., 2011; Almalki et al., 2021). This contrasts with a reported *Itln1* knockout mouse model in 129S5/SvEvBrd mice (Tang et al., 2010). Our *Tg*^*Vil1-Itln1*^ model represents a gut-specific gain of function model that closely mimics the expression pattern of ITLN1 in the human colon and IBD (Wang et al., 2020; Nonnecke et al., 2021; Nonnecke et al., 2022). Transgenic *Itln1* expression via the Villin-1 promoter led to ITLN1 expression in colonic goblet cells similar to that observed in humans (Nonnecke et al., 2021; Nonnecke et al., 2022; Wang et al., 2020). We used this human-like model to identify fecal bacteria that ITLN1 binds in vivo by developing a new quantitative ITLN1-seq approach to identify and quantify bacteria bound by endogenous ITLN1 in vivo. This contrasts with previous work that relied on ex vivo exposure of microbes to exogenous ITLN1 (Wesener et al., 2015).

We found that ITLN1 specifically binds a select group of fecal bacteria representing 0.5–1% of the microbial biomass in vivo. The most prominent member of the ITLN1-bound organisms was *A. muciniphila*, suggesting that ITLN1 targets a specific group of organisms within the intestinal lumen. *A. muciniphila* is a mucin-degrading bacterium (Derrien et al., 2004) that constitutes ∼1% of bacterial biomass in human stools (Derrien et al., 2008). As ITLN1 binding can differ among strains of the same bacterial species (Wesener et al., 2015), we verified ITLN1 binding to *A. muciniphila* isolated from mice and previously isolated human *A. muciniphila* (Derrien et al., 2004). ITLN1 binding to two independent *A. muciniphila* strains suggests that the glycan moiety that mediates the interaction is conserved across *A. muciniphila* strains. Identifying the specific moiety of *A. muciniphila* that binds to ITLN1 will require genetic modification of *A. muciniphila* and detailed biophysical studies. *A. muciniphila* has both protective and pathogenic effects, depending in part, on the degree of colonization of the host in multiple intestinal inflammatory models, including IL-10 colitis (Seregin et al., 2017), radiation and methotrexate intestinal injury, *Salmonella typhimurium* (Ganesh et al., 2013), and DSS colitis (Li et al., 2017; Bian et al., 2019). This cannot, however, explain our observations as we did not detect major differences in overall microbiota composition or *A. muciniphila* abundance in *Tg*^*Vil1-Itln1*^ mice.

However, the observation that ITLN1 is a core protein within mucus (van der Post et al., 2019) caused us to ask whether ITLN1 expression regulated the relationship between *A. muciniphila*, the mucus barrier, and IECs. ITLN1 overexpression reduced mucus layer thickness in a microbiota-dependent manner and facilitated the penetration of *A. muciniphila* into the inner mucus layer and its thinning. Together, these observations suggest that ITLN1-induced localization of mucolytic *A. muciniphila* within the mucus layer drives mucus thinning in *Tg*^*Vil1-Itln1*^ mice. Consequently, overexpression of ITLN1 (as occurs in patients with UC) in response to ER stress would be predicted to promote thinning of the mucus layer and facilitate the closer apposition of ITLN1-bound bacteria to IECs if mucolytic organisms such as *A. muciniphila* are present. This model suggests that ITLN1 expression must be tightly regulated and that either excessive or deficient ITLN1 expression differentially modifies disease by a process that, in part, reflects the local microbiota.

Further studies will be required to test this hypothesis and ultimately define how ITLN1, *A muciniphila*, and other microbiota members interact to modulate mucus layer thickness. This altered sensitivity to disease observed in *Tg*^*Vil1-Itln1*^ may be a consequence of mucus layer thinning, similar to the spontaneous colitis reported in *Muc2*^*−/−*^ mice (Van der Sluis et al., 2006) and in gnotobiotic *Il10*^*−/−*^ mice colonized with *A. muciniphila* (Seregin et al., 2017). However, we also found that ITLN1 promotes *A. muciniphila* uptake by macrophages, as previously reported for Bacillus Calmette–Guérin (Tsuji et al., 2009). This suggests that ITLN1 may enhance innate immune activation by promoting bacterial phagocytosis in addition to promoting mucus thinning. Although no ITLN1 receptor in immune cells has been identified, recent reports that the integrin receptors αvβ3 and αvβ5 (Lin et al., 2021) and adiponectin receptor-1 (Kobayashi et al., 2022) are potential ITLN1 receptors suggest that these proteins may contribute to increased phagocytosis of ITLN1-coated bacteria. However, further investigations are needed to extensively characterize the binding and signaling of ITLN1 in the context of interaction with bacterial targets and phagocytosis through these or other receptors.

Our findings suggest novel mechanisms by which UPR-regulated ITLN1 may affect the host’s susceptibility to intestinal inflammation in the colon that could act in addition to other mechanisms known to mediate abnormal inflammatory responses in IBD with colonic involvement (Caruso et al., 2020). We propose that ITLN1, through direct spatial regulation of a mucus-degrading microbe, makes the host susceptible to colitis. This mechanism suggests that UPR-mediated induction of excessive ITLN1 production in a host that possesses an ITLN1-binding bacterium with mucolytic properties may also predispose to immunopathology associated with enteropathogens. Together, this work provides a new perspective for understanding how a UPR-regulated protein can lead to the generation of inflammation in the colon.

## Materials and methods

### Mice

C57BL6 mice with deletion of *Xbp1* in epithelial cells (*Xbp1*^*ΔIEC*^) mice have been previously described (Kaser et al., 2008; Adolph et al., 2013; Grootjans et al., 2019). *Rag1*^−/−^ mice (Mombaerts et al., 1992) were obtained from the Jackson Laboratory (#002216; JAX stock). *Itln1*^*em1*(*IMPC*)*Wtsi*^ (*Itln1*^*−/−*^) mice were generated by endonuclease-mediated deletion of exon 4 using CRISPR-Cas9 technology at the Wellcome Trust Sanger Institute by a previously described approach (Pham et al., 2014; Cader et al., 2016). *Tg*^*Vil1-Itln1*^ mice expressing *Itln1* under the control of the 9 kb Villin-1 promoter (Pinto et al., 1999) were generated by cloning *Itln1* cDNA downstream of the Villin-1 promoter and upstream of the bovine growth hormone polyadenylation sequence and excising the Villin-1 promoter, *Itln1* cDNA sequence, and bovine growth hormone polyadenylation sequence by restriction digestion and injection into C57BL/6 embryos by the Harvard Genome modification Facility. *Itln1*^*−/−*^ and *Tg*^*Vil1-Itln1*^ mice on a C57BL/6 background were derived to GF conditions by Taconic Bioscience using their GF rederivation service. Briefly, *Itln1*^*−/−*^ and *Tg*^*Vil1-Itln1*^ males and wild-type female mice from our colony were used as sperm and oocyte donors, respectively, for in vitro fertilization and implantation in pseudopregnant recipient females housed in Taconic’s Gnotobiotic Facility. Pups were screened for GF status, and upon confirmation of GF status, they were transferred to the Massachusetts Host Microbiome Center at Brigham and Women’s Hospital, where they were housed under GF conditions or monocolonized with *A. muciniphila,* as previously described (Lavin et al., 2018). All mice were maintained in a SPF or gnotobiotic environment at Brigham and Women’s Hospital, according to institutional guidelines and the approval of relevant authorities.

Mice were screened by quantitative PCR (qPCR) of genomic DNA using Transnetyx. Mice were analyzed at 7–17 wk of age unless otherwise indicated. For all experiments, sex- and age-matched littermates were used as controls. Experimental groups were generated by heterozygous mating under SPF conditions in the *Itln1*^*−/−*^ colony or hemizygous mating in the *Tg*^*Vil1-Itln1*^ colony. Wild-type littermates from the *Itln1*^*−/−*^ colony are designated as wt and from the *Tg*^*Vil1-Itln1*^ colony as WT in the figures. *Rag1*^*−/−*^ mice were crossed to *Tg*^*Vil1-Itln1*^ mice to generate *Rag1*^*−/−*^*Tg*^*Vil1-Itln1*^ mice. *Rag1*^*−/−*^*Tg*^*Vil1-Itln1*^ mice and *Rag1*^*−/−*^ littermate controls were generated by hemizygous mating. Under GF conditions, as the maternal microbiota is not a concern, *Itln1*^*−/−*^ were generated by homozygous breeding.

### Mouse small intestinal organoids

Crypts were isolated from mouse small intestine by EDTA-based Ca^2+^/Mg^2+^ chelation, and intestinal organoids were cultivated as described (Stengel et al., 2020). In brief, the small intestine was removed and cut longitudinally. Intestinal pieces were incubated in PBS supplemented with 10 mM EDTA for 10 min with intermittent shaking. The supernatant was removed and PBS–EDTA solution was added. This procedure was repeated four times. The crypt suspension was passed through a 100-μm strainer and centrifuged at 400 ×*g*. Epithelial crypts were resuspended in Matrigel (BD Bioscience), embedded in 24-well plates, and cultivated in IntestiCult Organoid Growth Medium (STEMCELL) or ENR (EGF/Noggin/R-Spondin) media (Sato et al., 2009). The mouse models for the small intestine organoids from *Atf6*^*tg*^ mice and wild-type controls were already described (Stengel et al., 2020; Coleman et al., 2018). The medium was changed at least twice per week. Wild-type organoids were treated after 7 d of cultivation with 10 ng/µl tunicamycin for 24 h. *Atf6*^*tg*^ organoids and their wild-type controls were treated with 20 ng/µl for 24 h. UPR Inhibitors were added after 5 d of culture at the following concentrations in the presence of 10 ng/µl tunicamycin: 24 µM 4μ8c (Ghosh et al., 2014), 0.06 µM GSK2606414 (Guthrie et al., 2016), and 20 µM PF-429242 (Lebeau et al., 2018). Cells were harvested for RNA extraction after 24 h.

### Caco-2 cells

Caco-2_BBe_ cells (Kuo et al., 2019) were cultured in Dulbecco’s Modified Eagle Media (Corning) containing 10% FCS (Atlanta Biological; DMEM + 10% FCS) until 80% confluency under standard conditions. Cells were plated at 1 × 10^5^ cells in 12-well plates and 24 h later treated with vehicle and 10 ng/µl tunicamycin alone or in the presence of 60 µM 4μ8c, 3 µM GSK2606414, or 20 µM PF-429242 for 24 h before harvesting.

### RNA isolation and RT-qPCR

RNA from colon, organoids, primary human macrophages, or Caco-2 cells was extracted and purified using the RNeasy Plus Mini Kit (Qiagen) and from sorted LP macrophages using the RNAeasy Plus Micro Kit (Qiagen) as previously described (Gensollen et al., 2021). cDNAs were synthesized using SuperScript VILO IV reverse transcriptase (Life Technologies), Maxima H Minus First Strand cDNA Synthesis kit (Thermo Fisher Scientific), or oligo(dT) primers and Superscript IV (both Invitrogen, Thermo Fisher Scientific). Real-time RT-PCR was performed using AzuraQuant Green Fast qPCR Mix LoRox (Azuraquant) on a CFX96 Real-Time System (Bio-Rad) or using TaqMan Gene Expression Master Mix (Applied Biosystems) on the 7900HT Fast Real-Time PCR System (Applied Biosystems) or on an ABI Quant Studio 7 qPCR instrument (Thermo Fisher Scientific) using PowerUp SYBR Green Master Mix (Thermo Fisher Scientific). Values were normalized to *GAPDH,* and relative expression was calculated using the 2−ΔΔCT method (Livak and Schmittgen, 2001). Primers used for qPCR are available in Table S2.

### Luciferase experiment

1 × 10^5^ human embryonic kidney 293 T cells per well were plated in 48-well plates in DMEM + 10% FCS on day 0. 0.25 µg spliced XBP1 (XBP1-U, # 63679; Addgene), unspliced XBP1 (XBP1-s, #63680; Addgene), or control expression plasmid was transfected to cells with 0.25 µg *ITLN1* promoter report plasmid (#NEG-PG04; Genecopoeia) using Lipofectamine 3000 reagent (Invitrogen), as recommended by the manufacturer's instruction on day 1. On day 3, The reporter gene activities were measured by Secrete-Pair Dual Luminescence Assay Kit according to the manufacturer’s instructions (#LF031; Genecopoeia).

### Laser capture microdissection (LCM) of small intestinal crypts

Epithelial cells from the Paneth cell area in small intestinal crypt bases were harvested by LCM from germ-free *Xbp1*^*ΔIEC*^ mice using an Arcturus PixCell II system and CapSure HS LCM caps (Arcturus). Total RNA was prepared from captured cells using the Arcturus PicoPure RNA Isolation Kit as previously described (Vaishnava et al., 2008).

### Bulk RNA sequencing (RNA-seq) preparation and analysis

Libraries from LCM small intestinal crypts were prepared on a BioMek workstation (Beckman Coulter), including ribo-depletion and paired-end sequenced on the Illumina HiSeq2000 platform to generate 50-bp paired-end reads (uploaded to the Gene Expression Omnibus [GEO] accession number GSE175749). The FASTQ raw data were uploaded to Partek Flow (partek, building version: 10.0.21.0201). In Partek Flow, reads were trimmed by quality score and aligned to *Mus musculus* genome assembly GRCm38 (mm10) using STAR (v2.7.3a) followed by gene counting using HTSeq (v0.11. 0). Differentially expressed genes were quantified by DeSeq2, and significant genes were identified after correction for multiple comparisons using a false discovery rate (FDR) step-up <0.05.

Colonic bulk RNA-seq data were downloaded from GEO (accession number GSE128682). Raw gene counts were normalized by estimated size factors through DESeq2 v1.28.1. *apeglm* (v.1.10.0) was used as a shrinkage estimator. Hallmark pathway gene sets were downloaded from BROAD Molecular Signatures Database (version 6.2). The sum of the hallmark UPR pathway genes was used as normalized counts of the Hallmark UPR. Samples were matched to conditions according to the described overall design of the study (Taman et al., 2017). P values were used as calculated through DESeq2 v v1.28.1 using the Wald-test and corrected for multiple testing by the method of Benjamini and Hochberg (Love et al., 2014; Zhu et al., 2018).

### Single-cell RNA-seq (scRNA-seq) analysis

Epithelial scRNA-seq data were obtained from GEO (GSE116222), and goblet cell clusters were identified and subset as previously described (Parikh et al., 2019). Hallmark pathway gene sets were downloaded from BROAD Molecular Signatures Database (version 6.2). To score individual goblet cells for pathway activities, we used the R package AUCell (Aibar et al., 2017). Briefly, for each cell, expression matrix was used to compute gene expression rankings in each cell with the AUCell_build Rankings function with default parameters. Hallmark UPR pathway genes were then used to score each cell, where for each cell area-under-the-curve (AUC) values were computed (AUCell_calcAUC function) based on gene expression rankings, where AUC values then represent the fraction of genes within the top-ranking genes for each cell that are defined as part of the pathway gene set. Next, we fit a generalized negative binomial linear model (Zhang, 2018) to test whether *ITLN1* expression depended on UPR AUC values, blocking for individual donor effects in single-cell data, as well as cellular gene detection rate, as AUC values were highly correlated with the overall number of genes detected per cell.

### Immunohistochemistry

Intestinal sections were deparaffinized in xylene and rehydrated in graded ethanol to distilled water. For immunohistochemistry, endogenous peroxidase activity was blocked using 3% hydrogen peroxide in distilled water for 10 min. After heat-mediated antigen retrieval in 10 mM citrate buffer (pH 6.0), non-specific antibody binding sites were blocked (Animal-Free Blocking Solution, Cell Signaling Technology). Sections were incubated overnight at 4°C with rabbit anti-mouse ITLN1 (PAA933Mu01; Cloud Clone) at 1:400, rabbit anti-Chromogranin A (ab15160; Abcam) at 1:400, rabbit anti-DCLK1 (ab 37994; Abcam) 1:50, or sheep anti-human ITLN1 (AF4254; R&D Systems) 1:400. After thorough washing of the sections, an HRP-conjugated anti-rabbit polymer (SignalStain Boost IHC Detection Reagent, Cell Signaling Technology) or ImmPRESS HRP Horse Anti-Goat IgG Polymer Reagent (MP-7405; Vector Biolabs) that crossreacts with sheep primary antibodies was applied, and targets were visualized by 3,3′-diaminobenzidine-tetrahydrochloridedihydrate (Cell Signaling Technology). Sections were counterstained with hematoxylin. Small intestine samples from patients with IBD that were classified as GRP78 (−) and GRP78 (+) as previously described (Deuring et al., 2014) were stained for ITLN1 (#2019P002243; Institutional Review Board). Staining was scored blindly in Paneth cells on a scale from 0 to 2 by one of the authors (G.M. Fuhler).

### Isolation of LP leukocytes

The procedure was performed as described before (Grootjans et al., 2019). Briefly, colon was removed after euthanasia, following the removal of mesentery, fat, and intestinal content. Intestines were opened longitudinally and cut into 1–2 cm pieces. Samples were then placed into a 50-ml tube with 20 ml of HBSS 2 mM EDTA (Thermo Fisher Scientific) and incubated for 30 min at 37°C and 250 rpm on a shaking incubator twice. The tissue pieces were then collected, washed once in HBSS, and placed into a new 50-ml tube with digesting medium. Samples were incubated for 45 min at 37°C and 250 rpm. Then samples were filtered through a 100-μm cell strainer, washed with PBS 2% FBS and 2 mM EDTA, filtered through a 40-μm cell strainer, and washed again. Then cells were ready for analysis.

### Eukaryotic cell flow cytometry

Samples from the colon mucosa and large intestine luminal content were isolated as previously described (Grootjans et al., 2019). Single-cell suspensions were incubated with anti-mouse CD16/32 (clone 93; Biolegend) and counting beads (Spherotech) for 10 min at 4°C before staining. Cells were incubated in an antibody cocktail in PBS 2% FBS and Fixable Viability Dye (eBioscience), 2 mM EDTA for 30 min at 4°C. When required, intracellular staining was performed using a FOXP3 staining kit (eBioscience). After staining, cells were washed two to three times in PBS 2% FBS, 2 mM EDTA, and acquired on Cytoflex S (Beckman Coulter) or sorted using BD FACS Aria II (BD Biosciences) in RLT buffer (Qiagen) as previously described (Gensollen et al., 2021). Data were analyzed using FlowJo software v10 (BD Biosciences). Antibodies used for staining are in Table S3, and AccuCount Fluorescent particles 5.0–5.9 µm (Spherotech) were used for counting following manufacturer instructions.

### DNA extraction and 16S rRNA gene sequencing for analysis of microbial communities

Samples from the colon mucosa and large intestine luminal content of *Itln1*^−/−^ and *Tg*^*Vil1-Itln1*^ mice were isolated as previously described (Staubach et al., 2012). Bead beating using Lysing Matrix E tubes (MP Biomedical) was used prior to extraction to ensure cell lysis. The samples were extracted with the Qiagen Allprep DNA/RNA kit according to the manufacturer’s protocol. The V1-V2 region of the 16S rRNA gene was amplified according to the conditions described (Rausch et al., 2016) and was sequenced with 250 bp paired reads on the Illumina MiSeq platform at the Max Planck Institute for Evolutionary Biology.

Sequences were assigned to each sample by exact matches to multiplex identifier sequences and processed with the *dada2* R package (v1.16.0; Callahan et al., 2016). In brief, raw sequences were trimmed and quality-filtered with a maximum of two “expected errors” allowed in a read. Next, the paired sequences were merged, and chimeras were removed before assigning taxonomy using the Ribosomal Database Project training set 16. Samples were rarefied to a sequencing depth of 10,000 reads for all downstream analyses. Classifications with low confidence at the genus level (<0.8) were grouped in the arbitrary taxon “unclassified\_group”. Alpha (Shannon, Chao) and beta (Bray–Curtis) diversity were analyzed using the *phyloseq* R package (v1.32.0; McMurdie and Holmes, 2013). Differences in alpha diversity according to genotype were tested using a linear mixed model with the Shannon or Chao index as an outcome variable, genotype and sex as fixed effects, and dam identifier as a random effect. The *Vegan* package in R (v2.5-7) was used for analysis of dissimilarity using a constrained analysis of principal coordinates (“capscale”), a hypothesis-driven ordination that restricts the separation of the communities on the variable tested (Anderson and Willis, 2003), for which the “anova.cca” function was used to determine significance. Differentially abundant taxa between groups were determined with the *IndVal.g* function of the *multipatt* command in the *IndicSpecies* R package (Cáceres et al., 2010) with 10,000 permutations. Only taxa present in 25% of the samples were used for the IndicSpecies analysis. P values were corrected for multiple testing using FDR correction (Benjamini and Hochberg, 1995).

### Bacterial flow and ITLN1-seq

Bacterial flow was performed as described previously (Koch et al., 2016; Grootjans et al., 2019; Ansaldo et al., 2019) in ITLN1 binding buffer (20 mM Hepes [7.4], 150 mM NaCl, 10 mM CaCl2, 0.1% BSA, and 0.05% Tween-20; Wesener et al., 2015) with EDTA-free protease inhibitors (Roche). Briefly, fresh fecal pellets were homogenized in ITLN1 binding buffer with EDTA-free protease inhibitors and centrifuged at 50 ×*g* for 15 min at 4°C to remove large nonbacterial particles. The supernatant containing bacteria was transferred to a fresh tube, washed in ITLN1 binding buffer, centrifuged at 8,000 ×*g* for 5 min, and resuspended again in ITLN1 binding buffer. ITLN1 staining was performed by incubating the resuspended bacteria with 18 μg/ml biotin-conjugated anti-ITLN1 antibody (AF4254; R&D Systems) for 60 min at 4°C. Samples were washed and incubated with Streptavidin PE-Cy7 (eBioscience) 1:400 or Streptavidin PE (eBioscience) 1:200 for 20 min, followed by washing and resuspension in binding buffer with SYBR Green (Thermo Fisher Scientific) before flow cytometric analysis (Cytoflex S, Beckman Coulter) or sorting of the ITLN1 negative (ITLN1−) and ITLN1 positive (ITLN1+) fraction (SH800 FACS Cell Sorter, Sony). Bacteria were identified as SYBR high (SYBR^hi^) events as described previously (Koch et al., 2016; Grootjans et al., 2019; Ansaldo et al., 2019; Fig. S3 Q). After sorting, sample processing and 16S rRNA gene sequencing was performed at the Massachusetts Host-Microbiome Center. Briefly, DNA was extracted from presorted and postsorted samples using Quick-DNA Fecal/Soil Microbe Miniprep Kit (Zymo Research). A multiplexed amplicon library covering the 16S rDNA gene V4 region was generated from DNA-extracted samples, and reads were generated on the MiSeq instrument from the amplicon library as previously described (Fujisaka et al., 2018). Paired-end 16S rRNA V4 reads were trimmed for quality (target error rate <0.5%) and length (minimum 200 bp) using Trimmomatic (Bolger et al., 2014) merged using FLASH (Magoč and Salzberg, 2011), and quality screened using QIIME (Bolyen et al., 2019). Spurious hits to the PhiX control genome were identified using BLASTN and removed. Passing sequences were trimmed of primers, evaluated for chimeras with UCLUST (de novo mode in QIIME), and screened for mouse-associated contaminants using Bowtie2 (Langmead and Salzberg, 2012), followed by a more sensitive BLASTN search against the GreenGenes 16S rRNA database. Chloroplast and mitochondrial contaminants were detected and filtered using the Ribosomal Database Project classifier (Wang et al., 2007) with a confidence threshold of 50%. High-quality 16S rRNA sequences were assigned to a high-resolution taxonomic lineage using Resphera Insight (Shaikh et al., 2021; Drewes et al., 2017). Alpha and beta diversity measures were calculated using QIIME. Downstream statistical analysis utilized R (v3.5.3) with log-transformed Welch’s *t* tests for differential abundance assessment of individual taxonomic features and PERMANOVA for comparisons of total community composition (adonis package). Multiple hypothesis testing correction employed the FDR (Benjamini and Hochberg, 1995).

### Bacterial cultures

*Streptococcus pneumoniae* (Klein) Chester serotypes 8 (6308; ATCC) and 43 (10343; ATCC) were obtained from the ATCC. *S. pneumoniae* strains were grown on Brain Heart Infusion Agar or Broth. *S. pneumoniae* were grown at 37°C under 5% carbon dioxide gas. During liquid culture, cells were in stationary phase. *A. muciniphila* (BAA-835; ATCC) was obtained from ATCC or isolated from our mice. To isolate *A. muciniphila* in our SPF colony, stools were collected in sterile tubes containing two to three fecal pellets and kept at −80 until processed. The samples were placed in an anaerobic chamber with an atmosphere of 10% hydrogen, 10% carbon dioxide, and 80% nitrogen. 500 μl of PBS prereduced with 0.05% cysteine hydrochloride was added to each tube. The samples were vortexed to create a homogenous slurry. Serial 10-fold dilutions of the slurry were made in PBS to 10^−6^, and 100 μl of the original sample and dilutions were plated onto mucin agar plates prepared as described in Ansaldo et al. (2019). The agar plates were incubated for up to 5 d. Colonies were subcultured onto Brucella agar with hemin, vitamin K, and mucin agar plates. Colonies consistent with the phenotypic characteristics of *A. muciniphila* were Gram-stained, and their identity was confirmed by Sanger sequencing. Briefly, the full 16S rRNA gene of the obtained isolate was amplified using universal 16S primers (27F and 1492R), sequenced (genewiz), and revealed to be identical to the 16S rRNA gene for the *A. muciniphila* strain ATCC BAA-835.

### ITLN1 binding assay

To analyze recombinant human ITLN-1 (rITLN1) binding to the bacterial cell surface by flow cytometry, we harvested bacteria by centrifugation, washed them with PBS, and fixed them in 1% formaldehyde in PBS for 30 min on ice as previously described (Wesener et al., 2015). Cells were resuspended in ITLN1 binding buffer and frozen at −80°C until further use. Bacteria were stained with 15 µg/ml rITLN-1 (R&D) as previously described (Wesener et al., 2015), and coating by ITLN1 was detected as described for ITLN1-seq.

### Monocolonization of GF mice with *A. muciniphila*

6–7-wk-old mice GF wild-type, *Itln1*^*−/−*^, and *Tg*^*Vil1-Itln1*^ mice matched by sex were gavaged with 2 × 10^6^ *A. muciniphila* isolated from our SPF mice in sterile isolators as described (Lavin et al., 2018). Mice were collected for methacarn embedding 21 d postinoculation. Monocolonization was confirmed by culture of feces at 7 and 21 d after inoculation, and *A. muciniphila* in stools was quantified by qPCR at collection.

### *A. muciniphila* quantification by qPCR

DNA was extracted from stool or colonic tissue lysates using QIAamp DNA Stool Mini Kit (Qiagen). qPCR was performed using AzuraQuant Green Fast qPCR Mix LoRox (Azuraquant) and a CFX96 Real-Time System (Bio-Rad). Primers against a region of the *A. muciniphila* 16S rRNA gene were used. Genome equivalents per gram were calculated by comparing cycle threshold values to a dilution series of *A. muciniphila* genomic standard (Sigma-Aldrich) and normalizing for the amount of input (weight) as previously described (Ansaldo et al., 2019).

### Methacarn embedding

The entire colon was dissected with stool pellets in place. Colons were embedded in methacarn as previously described (Johansson and Hansson, 2011). Briefly, colons were left in methacarn for 24 h, followed by processing and embedding in paraffin at the Beth Israel Deaconess Medical Center histology core with methanol, ethanol, and xylene washes in the absence of water. Sections from the distal two-thirds of the colon were cut every 50 µm only if the fecal pellet was in place. Both genotypes and their age-matched littermates were processed within the same week and in the same histology core and with the same reagents.

### Methacrylate embedding

Samples from the distal third of the colon were embedded in methacrylate with the stool pellet in place, as previously described (Hasegawa et al., 2017; Welch et al., 2017), using an EtOH dilution series for gentle dehydration before transferring to acetone. Sections were cut dry to 5-µm thickness using glass knives on a rotary microtome and then transferred onto a drop of water on a slide.

### FISH and immunofluorescence

FISH was performed using custom synthesized probes (biomers.net GmbH and Integrated DNA Technologies) dually labeled (5′ and 3′) with Atto 550 (EUB338 I & III = Eubacteria probes; Daims et al., 1999; Amann et al., 1990) or Texas Red X (Muc1437 = *A. muciniphila* probe; Table S4; Derrien et al., 2008). For paraffin slides, dewaxing was performed as previously described (Johansson and Hansson, 2011). Sections were incubated in hybridization buffer (0.9 M NaCl, 0.02 M Tris, pH 7.5, 0.01% SDS, 20% HiDi formamide, 2 µM probe) at 46°C for 4 h. After hybridization, samples were washed at 48°C for 15 min in wash buffer (0.215 M NaCl, 0.02 M Tris, pH 7.5, 0.005 M EDTA). After washing with PBS, sections were treated with a blocking solution (Cell Signaling, Animal Free) for 30 min at 4°C. For methacrylate samples, sections were incubated with 1:400 dilution of anti-mouse ITLN1 antibody (PAA933Mu01; Cloud Clone) overnight at 4°C. After washing in PBS, a 1:1,000 dilution of secondary antibody (Alexa Fluor 647, goat anti-rabbit; Thermo Fisher Scientific) was added. Slides were incubated for 2 h at 4°C. After washing in PBS and air drying, sections were counterstained with DAPI (1 µg/ml; Thermo Fisher Scientific) and WGA (20 µg/ml; Alexa Fluor 488 Conjugate, Thermo Fisher Scientific) at room temperature for 30 min. After rinsing in ice-cold water, slides were mounted using ProLong Gold antifade (Thermo Fisher Scientific). For methacarn samples, sections were then incubated with 1:500 dilution of Mucin-2 H-300 (sc15334; Santa Cruz) and 1:100 γ-actin conjugated AF790 (sc65638; Santa Cruz) overnight at 4°C. After washing in PBS, a 1:1,000 dilution of secondary antibody (Alexa Fluor 647, goat anti-rabbit; Thermo Fisher Scientific) was added. Slides were incubated for 2 h at 4°C. After washing in PBS and air drying, sections were counterstained with DAPI (1 µg/ml; Thermo Fisher Scientific) and WGA (20 µg/ml; Alexa Fluor 488 Conjugate, Thermo Fisher Scientific) at room temperature for 30 min. After rinsing in ice-cold water, slides were mounted using ProLong Gold antifade (Thermo Fisher Scientific).

### Image acquisition and processing

For methacrylate-fixed tissues, images were acquired at the Forsyth Institute Advanced Microscopy Core Facility (RRID:SCR_021121) using an LSM 780 (Carl Zeiss) microscope equipped with a 32-channel multianode spectral detector with 8.9-nm channel widths. Images were acquired with a 40× 1.4 NA Plan-Apochromat objective at a pixel size of 0.104 × 0.104 μm. Three-dimensional image stacks were acquired as a series of at most 10 optical sections with a *z*-step of 0.49 μm. Image positions were selected for integrity of the section and the mucus layer as well as presence of bacteria in the fecal content. Each field of view was imaged sequentially, first using a 594 nm laser line and then using simultaneous excitation with 405, 488, 561, and 633 nm laser lines and a triple dichroic beam splitter. Linear unmixing of the fluorescence emission was performed using Zeiss ZEN software or the nonlinear least-squares function in MATLAB. For the image stacks acquired with the 594 nm laser line, a 3 × 3 median filter was applied, followed by linear unmixing using reference emission spectra. For the image stacks acquired with four laser lines simultaneously, a maximum intensity projection was produced and a 3 × 3 median filter was applied followed by linear unmixing using reference emission spectra. Reference spectra were collected using the same laser lines and dichroic filters as the experimental acquisitions and by imaging single-labeled specimens.

For methacarn-fixed tissues, images were collected using a Leica DM4000 microscope with 40× NA 0.6 HCX PL FLUOTAR objective (Leica) with CMOS camera (Hamamatsu), motorized emission filter wheel (Ludl), xyz-motorized stage (Ludl), five-channel Aura light engine (Lumencor), and a multichannel dichroic matched to single band emission filters (Semrock) all controlled by Metamorph 7.9 (Molecular Devices). Spillover from the nuclei staining with DAPI into the 565–615 nm channel was compensated using the image calculator function in ImageJ (Schindelin et al., 2012).

### Measurement of the inner mucus layer thickness

For each animal, three transverse sections of the fecal pellet cut at least 50 µm apart were examined for methacrylate-fixed tissues and six transverse sections for methacarn-fixed tissues. For each section, three fields of view were analyzed. To assess mucus thickness, we used ImageJ (Schneider et al., 2012; Schindelin et al., 2012). Four measurements per field of view were taken at points at least 50 μm apart as a conservative estimate of the distance at which independent measurements can be obtained (Earle et al., 2015). The researcher (P. Griebel) measuring the mucus layer was blinded to the genotypes.

For methacarn samples, the inner mucus layer was defined as a well-organized stratified MUC2 lamellar appearance, delimitated by the microbiota on the luminal side under SPF conditions or the *A. muciniphila* signal in monocolonized mice (Johansson et al., 2008; Johansson et al., 2015), and γ-actin, a marker of the apical epithelial cell lining (Kaji et al., 2020). Under GF conditions, the inner mucus layer was defined as a well-organized stratified MUC2 lamellar layer (Johansson et al., 2008; Johansson et al., 2015; Bergstrom et al., 2020) above γ-actin. In methacrylate samples, the inner mucus layer was defined as the WGA stratified layer between the bacterial biomass and the epithelial border detected by autofluorescence in DAPI (Earle et al., 2015).

### Proximity analysis of *A. muciniphila* localization in the inner mucus layer

To quantify the spatial distribution of *A. muciniphila* regarding the mucus layer, we used methacrylate-fixed tissues to preserve better the three-dimensional structure of the intestinal microbial communities (Welch et al., 2017; Hasegawa et al., 2017). ImageJ was used to binarize Eubacteria and *A. muciniphila* channels using auto local thresholding with the Bernsen method (Nichele et al., 2020) as well as size (0.1–1 µm^2^) and circularity (0.6–1.0) filters. Proximity analysis was carried out using the linear-dipole algorithm in DAIME (Daims et al., 2006) using the outlined mucus edge closest to the epithelium as the reference. The average pair correlation value for each distance point was calculated from average measurements for each mouse with four mice per genotype (3 field of view/section × 3 sections/animal × 4 animals/genotype = 36 field of view per genotype). Due to low bacterial counts closer to the mucus edge, individual zero values due to no observed bacteria at the corresponding distance and final average values with SD = 0 were excluded.

### DSS colitis

Sex- and age-matched littermates received 2 or 3% DSS (MP Biomedicals) in drinking water for 7 d and then regular water thereafter. Weight was recorded daily. Mice were sacrificed between 2–9 d after DSS treatment for histological and immunological assessment.

### Histopathological analyses of DSS colitis

Postmortem, the entire colon was excised. Swiss rolls (Moolenbeek and Ruitenberg, 1981) were prepared starting with the distal part, keeping the luminal side facing outward. The entire specimen was fixed in 4% formalin. Paraffin sections were cut and stained with H&E. A semiquantitative composite scoring system was used for the assessment of intestinal inflammation, calculated as a sum of four histological subscores as follows: mononuclear cell infiltration (0: absent; normal sparse lymphocytic infiltration, 1: mild; diffuse increase in LP, 2: moderate; LP increased with basal localization aggregates displacing crypts, 3: severe; LP with submucosal infiltration), crypt hyperplasia (0: absent, 1: mild, 2: moderate, 3: severe), epithelial injury/erosion (0: absent, 1: mild; crypt dropout or surface epithelial damage without frank erosion or ulceration, 2: moderate; focal ulceration, 3: severe; multifocal or extensive ulceration), and polymorphonuclear cell infiltration (0: absent, 1: mild; LP only, 2: moderate; LP infiltration with cryptitis or crypt abscesses, 3: severe; sheet-like or submucosal infiltration). Scores were multiplied by a factor based on the extent of the inflammation. Extent factor was derived according to the fraction of bowel length involved by inflammation: 1, <10%; 2, 10–25%; 3, 25–55%; and 4, >55%. The score was assessed by an expert gastrointestinal pathologist (J.N. Glickman) who was blinded to the genotype and experimental conditions of the samples.

### Intestinal explant culture

For intestinal explant culture, two whole-layer punches cut by Tru-Punch Sterile Disposable Biopsy Punch 6 mm (Sklar) were incubated in 24-well tissue culture plates containing 500 μl of RPMI 2% FBS (Atlanta Biologicals), 1% HEPES (Corning), and antibiotic/antimycotic (Gibco) at 37°C and 5% CO_2_ for 24 h. Following the protocol, supernatants were stored at −80°C until further use. Cytokine expression levels were measured using BioLegend’s bead-based immunoassays following manufacturer instructions.

### Naive T cell colitis

Sex- and age-matched *Rag1*^*−/−*^ and *Rag1*^*−/−*^*Tg*^*Vil1-Itln1*^ littermates were injected intraperitoneally with 5 × 10^5^ naive CD4^+^ T cells isolated from splenocytes as previously described at 7 wk of age (Raju et al., 2020). Weight was recorded prior to animals being injected and once a week. Mice were monitored for disease progression and were sacrificed after 6 wk or if the weight loss was ∼15–20% of their original body weight. Colon lengths and weight were measured, and colonic weight for length was calculated as previously described (Ostanin et al., 2008)

### Clearance of *A. muciniphila* with tetracycline

Prior to DSS administration, *A. muciniphila* in stool samples was measured in mice by qPCR, confirming colonization. Mice were treated in drinking water with 3 g/liter of tetracycline diluted autoclaved water, pH 7.4, for 3 wk. The antibiotic in drinking water was changed twice a week (Ansaldo et al., 2019). Following treatment, animals were placed for 1 wk in water and were restricted and handled by one researcher (J.A. Tascon-Arcila). Quantification of *A. muciniphila* by qPCR in stool was repeated after the antibiotic course and after the DSS exposure showing undetectable levels of *A. muciniphila* on both occasions.

### Preparation of human monocyte-derived macrophages

Healthy volunteer blood cones were obtained from the NHS Blood and Transplant Bank, and peripheral blood mononuclear cells (PBMCs) were isolated through Histopaque 1077 (MilliporeSigma) density centrifugation. Macrophage preparation was performed as previously published (Flak et al., 2019). In short, PBMCs on 10-cm tissue culture plates were incubated in calcium- and magnesium-containing PBS at 37°C for 30 min, followed by washing with calcium- and magnesium-free PBS and incubation of adherent cells for 7 d in 20 ng/ml GM-CSF and 10% human serum-containing RPMI 1640 at 5% CO_2_ and 37°C. Cells were used on day 7 for the experiments below.

### Phagocytosis assays

*A. muciniphila* were stained using pHrodo Red Dye (Invitrogen, Thermo Fisher Scientific) as per the manufacturer’s recommendation, then incubated in RPMI 1640 containing 1% FCS and 1 nM human recombinant ITLN1 (R&D Systems, Bio-Techne Ltd.) or 1% FCS-containing RPMI 1640 alone, for 2 h at 37°C.

Monocyte-derived macrophages on 96-well plates (4 × 10^4^ cells/well) were stained with Hoechst 33342 (Thermo Fisher Scientific) for 1 h at 37°C for cell nucleus visualization and washed with RPMI-1640. Labeled and ITLN1-coated or uncoated *A. muciniphila* (ratio 50:1 of bacterial cells:macrophages) were added to macrophages. Over time, the increase in pHrodo Red signal (indicating phagocytosis of bacterial cells by macrophages) was quantified using a Zeiss Cell Discoverer 7 high-content imaging system.

### Cytokine production by phagocytes

Human monocyte-derived macrophages were prepared as detailed above and, on day 7 plated onto 6-well tissue culture plates at 1.2 × 10^6^ cells/well. *A. muciniphila* were incubated in 1% FCS and 1 nM human recombinant ITLN1 (R&D Systems, Bio-Techne Ltd.)-containing RPMI 1640 or 1% FCS-containing RPMI 1640 alone, for 2 h at 37°C.

ITLN1-coated or uncoated bacterial cells were added to macrophages at a ratio of 50:1 of bacteria:macrophages and incubated at 37°C for 2 h. No bacteria were added to control cells. Macrophages were scraped into RLT buffer (QIAGEN) containing 1% 2-mercaptoethanol for RNA extraction and qPCR as described above.

### Statistical analysis

Statistical significance was determined as indicated in the figure legends. Differences were considered significant at P < 0.05. Data were analyzed using GraphPad Prism v9 (GraphPad Software), Partek flow, or R version 4.0.2.

### Online supplemental material

Fig. S1 shows that Intelectin-1 transcription is induced upon ER stress activation, and its absence or overexpression in intestinal epithelial cells does not affect colonic enteroendocrine cells, Tuft cells, and Goblet cells. Fig. S2 shows the baseline characterization of LP leukocytes in the *Itln1*^*−/−*^ and *Tg*^*Vil-Itln1*^ mice. Fig. S3 shows the baseline characterization of the microbiota of *Itln1*^*−/−*^ and *Tg*^*Vil-Itln1*^ mice. Fig. S4 shows *A. muciniphila* binding to ITLN1 in vitro, *A. muciniphila* quantification by qPCR, and additional methacarn and methacrylate fixed tissue imaging. Fig. S5 shows that ITLN1 overexpression worsens colitis in DSS-induced and naive T transfer-induced colitis model. Table S1 shows ASVs significantly enriched in the *Tg*^*Vil1-Itln1*^ or *Itln1*^*−/−*^ mice compared to wt littermates. Table S2 shows primers used for RT-qPCR experiments. Table S3 shows antibodies used for eukaryotic flow cytometry. Table S4 shows FISH probes used.

## Supplementary Material

Table S1 shows ASVs significantly enriched in the *Tg*^*Vil1-Itln1*^ or *Itln1*^*−/−*^ mice compared to wt littermates.

Table S2 shows primers used for RT-qPCR.

Table S3 shows antibodies used for eukaryotic flow cytometry.

Table S4 shows FISH probes.
