# Supplementary material for: Intelectin-1 binds and alters the localization of the mucus barrier–modifying bacterium Akkermansia muciniphila
Source: J Exp Med. 2022 Nov 22;220(1):e20211938. doi: 10.1084/jem.20211938 (PMC9683900; doi:10.1084/jem.20211938)
Supplement: Table S1 — shows ASVs significantly enriched in the TgVil1-Itln1 or Itln1−/− mice compared to wt littermates. [file JEM_20211938_TableS1.docx]

| Taxon | Indicator for | *P* value | AdjP-value* | Site |
| --- | --- | --- | --- | --- |
| ASV115 (unclassified *Bacteroidales*) | *Tg^Vil1-Itln1^* | 0.00017 | 0.0108 | Lumen |
| ASV31 (unclassified *Clostridiales*) | *Tg^Vil1-Itln1^* | 0.00008 | 0.0085 | Lumen |
| ASV33 (unclassified *Bacteroidales*) | *Tg^Vil1-Itln1^* | 0.00004 | 0.0085 | Lumen |
| ASV445 (unclassified *Bacteroidales*) | *Tg^Vil1-Itln1^* | 0.00008 | 0.0085 | Lumen |
| ASV75 (Genus *Alistipes*) | *Tg^Vil1-Itln1^* | 0.00012 | 0.0095 | Lumen |

Table S1. ASVs significantly enriched in the *Tg^Vil1-Itln1^* or *Itln1^-/-^* mice compared to wt littermates. *P values are corrected for multiple comparisons using the Benjamini-Hochberg method (F.D.R. (n_core ASVs_=317).
