# Supplementary material for: Intelectin-1 binds and alters the localization of the mucus barrier–modifying bacterium Akkermansia muciniphila
Source: J Exp Med. 2022 Nov 22;220(1):e20211938. doi: 10.1084/jem.20211938 (PMC9683900; doi:10.1084/jem.20211938)
Supplement: Table S2 — shows primers used for RT-qPCR. [file JEM_20211938_TableS2.docx]

| **Organism** | **Target** | **Sequence or reference** |
| --- | --- | --- |
| *A. muciniphila* | *16S rRNA* (Ansaldo et al., 2019) | AGTATCGAAAGATTAAAGCAGCAATGC  TCTTGTGGTACTATCTTTTTAATTTGCT |
| *Homo sapiens* | *GAPDH* (Nabokina et al., 2014) | GTCTCCTCTGACTTCAACAGCG  ACCACCCTGTTGCTGTAGCCAA |
| *Homo sapiens* | *HSPA5* (PrimerBank) | CATCACGCCGTCCTATGTCG  CGTCAAAGACCGTGTTCTCG |
| *Homo sapiens* | *IL-6* | QT00083720 (QIAGEN) |
| *Homo sapiens* | *IL-10* | GACTTTAAGGGTTACCTGGGTTG  TCACATGCGCCTTGATGTCTG |
| *Homo sapiens* | *ITLN1* (PrimerBank) | AGGAAAGTGCAGCTGAGACT  GGAGACGAAGAACAGGTCCA |
| *Homo sapiens* | *TNF* | QT00029162 (QIAGEN) |
| *Mus musculus* | *Gapdh* | QT01658692 (QIAGEN) |
| *Mus musculus* | *Gapdh* | *Gapdh*_Mm99999915_g1 (ThermoFisher) |
| *Mus musculus* | *Hspa5* | *Hspa5*_Mm00517690_g1 (ThermoFisher) |
| *Mus musculus* | *Itln1* | *Itln1*_Mm04243906_g1 (ThermoFisher) |
| *Mus musculus* | *Muc2* | QT01060773 (QIAGEN) |
| *Mus musculus* | *Tnf* | QT00104006 (QIAGEN) |

**Table S2. Primers used for RT-qPCR**
