# Supplementary material for: Intelectin-1 binds and alters the localization of the mucus barrier–modifying bacterium Akkermansia muciniphila
Source: J Exp Med. 2022 Nov 22;220(1):e20211938. doi: 10.1084/jem.20211938 (PMC9683900; doi:10.1084/jem.20211938)
Supplement: Table S3 — shows antibodies used for eukaryotic flow cytometry. [file JEM_20211938_TableS3.docx]

| **Antigen** | **Clone** | **Vendor** | **Dilution** |
| --- | --- | --- | --- |
| Ly6C | Al-21 | BD | 800 |
| CD64 | X54-5/7.1 | RD | 300 |
| MHCII | M5/114.15.2 | eBiocience | 300 |
| SiglecF | E5-2440 | BD Biosciences | 300 |
| CD103 | M290 | BD Biosciences | 200 |
| eFluor506 | NA | Thermofisher | 1000 |
| CD11c | N418 | BD | 200 |
| CD45 | 104 | BD | 400 |
| CCR2 | 475301 | Fisher | 100 |
| Cd11b | M1/70 | BD | 400 |
| F4/80 | BM8 | Thermofisher | 200 |
| GATA-3 | TWAJ | Bioscience | 200 |
| NK1.1 | PK136 | Biolegend | 400 |
| CD45 | 30F-11 | Biolegend | 800 |
| CD3 | 17A2 | Biolegend | 300 |
| CD5 | 53-7.3 | Biolegend | 300 |
| CD19 | 6D5 | Biolegend | 300 |
| Ly6G | 1A8 | Biolegend | 300 |
| Eomes | DAN1 mag | Bioscience | 100 |
| eFluor506 | NA | Thermofisher | 1000 |
| IL7Ra | A7R34 | Biolegend | 200 |
| RORgt | B2D | eBioscience | 100 |
| Tbet | 4B10 | Biolegend | 200 |
| CD4 | RM4-5 | Biolegend | 800 |
| CD11b | M1/70 | Biolegend | 400 |
| NK1.1 | PK136 | Biolegend | 400 |
| Ly6G | 1A8 | BDB.D.iosciences | 200 |
| CD19 | 6D5 | Biolegend | 300 |
| MHCII | M5/114.15.2 | eBioscience | 400 |
| eFluor506 |  | Thermofisher | 1000 |
| CD8 | 53-6.7 | Biolegend | 400 |
| CD45 | 104 | BD | 400 |
| Siglec F | E5-2440 | BD Biosciences | *800* |

**Table S3. Antibodies used for eukaryotic flow cytometry**
