# Supplementary material for: Intelectin-1 binds and alters the localization of the mucus barrier–modifying bacterium Akkermansia muciniphila
Source: J Exp Med. 2022 Nov 22;220(1):e20211938. doi: 10.1084/jem.20211938 (PMC9683900; doi:10.1084/jem.20211938)
Supplement: Table S4 — shows FISH probes. [file JEM_20211938_TableS4.docx]

| **Name** | **Organism/Target** | **Sequence (5’ to 3’)** |
| --- | --- | --- |
| MUC1437 (Derrien et al., 2008) | *A. muciniphila* | CCTTGCGGTTGGCTTCAGAT |
| EUB338-I (Amann et al., 1990) | Conserved 16s region | GCTGCCTCCCGTAGGAGT |
| EUB338-III (Daims et al., 1999) | Conserved 16s region | GCTGCCACCCGTAGGTGT |

**Table S4. FISH Probes**
